# GenRCA: a user-friendly rare codon analysis tool for comprehensive evaluation of codon usage preferences based on coding sequences in genomes

**DOI:** 10.1186/s12859-024-05934-z

**Published:** 2024-09-27

**Authors:** Kunjie Fan, Yuanyuan Li, Zhiwei Chen, Long Fan

**Affiliations:** 1 Production and R&D Center I of LSS, GenScript (Shanghai) Biotech Co., Ltd., Shanghai, China; 2 Production and R&D Center I of LSS, GenScript Biotech Corporation, Nanjing, China

**Keywords:** Codon usage, Rare codon analysis, Protein expression, Gene design

## Abstract

**Background:**

The study of codon usage bias is important for understanding gene expression, evolution and gene design, providing critical insights into the molecular processes that govern the function and regulation of genes. Codon Usage Bias (CUB) indices are valuable metrics for understanding codon usage patterns across different organisms without extensive experiments. Considering that there is no one-fits-all index for all species, a comprehensive platform supporting the calculation and analysis of multiple CUB indices for codon optimization is greatly needed.

**Results:**

Here, we release GenRCA, an updated version of our previous Rare Codon Analysis Tool, as a free and user-friendly website for all-inclusive evaluation of codon usage preferences of coding sequences. In this study, we manually reviewed and implemented up to 31 codon preference indices, with 65 expression host organisms covered and batch processing of multiple gene sequences supported, aiming to improve the user experience and provide more comprehensive and efficient analysis.

**Conclusions:**

Our website fills a gap in the availability of comprehensive tools for species-specific CUB calculations, enabling researchers to thoroughly assess the protein expression level based on a comprehensive list of 31 indices and further guide the codon optimization.

**Supplementary Information:**

The online version contains supplementary material available at 10.1186/s12859-024-05934-z.

## Introduction

The Codon Usage Bias (CUB) refers to the non-random usage of synonymous codons encoding the same amino acid within a genome or set of genes [1]. It reflects the preference or bias in the selection of specific codons over others during translation. Various factors, including expression level, GC content, recombination rates, RNA stability, codon position, gene length, environmental stress, and population size, can influence CUB within and among species [2, 3]. Understanding CUB is important as it provides insights into evolutionary processes, gene expression regulation, protein folding, and adaptation to different environments [4–6]. CUB indices are effective tools to study the pattern of codon usage bias, allowing for straightforward and computationally efficient evaluations of species-specific codon usage, eliminating the requirement for extra experiments, providing valuable insights into the genetic characteristics of organisms.

Over the past few years, a number of measures have been proposed to quantify CUB. There exist some free websites that support the calculation of codon usage preferences, as outlined in Table 1. Among them, the first version of our publicly available website, GenScript Rare Codon Analysis, initially launched in 2008, has gained significant popularity with a high daily user visit count and over 300 citations by supporting 3 types of CUB indices and 17 species along with informative visualizations. As for other websites, they either only provide basic codon usage frequency distribution or support simply one index, which is not useful. Given that there is no universal index that fits all species, there is a significant need for a comprehensive platform that supports the calculation of multiple CUB indices for various species, enabling thorough evaluation of codon usage preferences for coding sequences.

**Table 1:** Description of existing rare codon analysis websites

URL	Content	Species	Batch	Download
GenRCA rare Codon Analysis Tool (genscript.com)	Codon usage frequency distribution 31 CUB indices	65	Yes	Yes
https://www.biologicscorp.com/tools/RareCodonAnalyzer	Codon usage frequency distribution 1 CUB index (CAI)	14	No	No
http://www.detaibio.com/tools/rare-codon-analyzer.html	Codon usage frequency distribution	14	No	Yes
E. coli codon usage analyzer (ucr.edu)	Codon usage frequency distribution	1	No	No
Rare Codon Caltor, Programmed by Edmund Ng (ucla.edu)	Frequency of codon occurrence	1	No	No
Rare Codon Search (bioline.com)	Searching for rare codons	6	No	No
http://www.bitgene.net/dna/rare_codon	Codon usage frequency distribution	1	No	No

“Batch” refers to whether the website supports batch processing of multiple sequences. “Download” denotes whether the website supports the download of analysis report

**Fig. 1:**
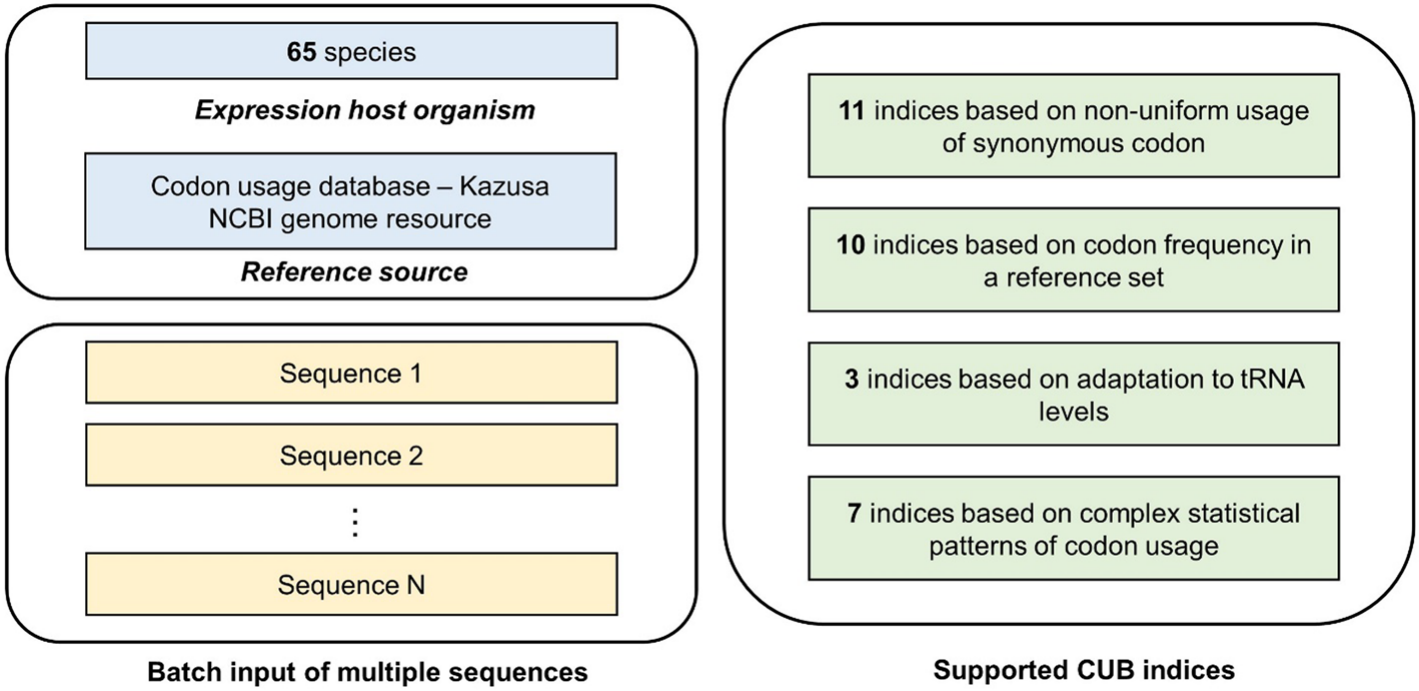
Overview of supported functionalities in GenRCA website. GenRCA includes the calculation of 31 CUB indices for 65 species and two reference sources, as well as the batch processing of multiple input sequences

Hence, in this study, we release GenRCA, a user-friendly website for all-inclusive rare codon analysis, which is freely available at https://www.genscript.com/tools/rare-codon-analysis. Compared to our previous version, the available number of codon preference indices is extended from 3 to 31 and the number of supported expression host species is increased from 17 to 65, enabling users to explore and compare codon usage biases across a wider range of species in a more comprehensive manner (Fig. 1). Furthermore, we introduced a new feature that enables batch processing of multiple gene sequences. With a number of supported indices and species, informative visualizations, and user-friendly interface, our website holds great potential to improve the overall convenience and accuracy of protein expression optimization (Table 2).

**Table 2:** List of 31 supported indices on our website

Category	Indices
Indices based on non-uniform usage of synonymous codon	RSCU (Relative Synonymous Codon Usage) [7]
	ENC (Effective Number of Codons) [8, 9]
	RCBS (Relative Codon Bias Strength) [10]
	DCBS (Directional Codon Bias Score) [11]
	CDC(Codon Deviation Coefficient) [12]
	MILC (Measure Independent of Length and Composition) [13]
	ICDI (Intrinsic Codon Deviation Index) [14]
	SCUO (Synonymous Codon Usage Order) [15, 16]
	Ew (Weighted Sum of Relative Entropy) [17]
	P (Codon Preference) [18]
	MCB (Maximum-likelihood Codon Bias) [19]
Indices based on codon frequency in a reference set of genes	CAI (Codon Adaptation Index) [20]
	FOP (Frequency of Optimal Codons) [21, 22]
	COUSIN (Codon Usage Similarity Index) [23]
	CBI (Codon Bias Index) [24]
	Dmean (Mean Dissimilarity-based Index) [25]
	RCA (Relative Codon Adaptation) [26]
	CUFS (Codon Usage Frequency Similarity) [27]
	B (Codon Usage Bias) [28]
Indices based on adaptation to the tRNA levels and their supply	tAI (tRNA Adaptation Index) [29]
	gtAI (Genetic tRNA Adaptation Index) [30]
	P2 index [31]
Indices based on complex patterns of codon usage	GC content [32]
	ENcp (Effective Number of Codon Pairs) [33]
	CPS (Codon Pair Score) [34, 35]
	Codon volatility [36]

COUSIN includes COUSIN18 and COUSIN59

GC content can be subdivided into GC, GC1, GC2, GC3

## Implementation

Our implemented rare codon analysis tool is provided as a free and user-friendly website. We manually collected almost all available CUB-related papers and re-implemented 31 proposed methods in Python programming language. We also aggregated reference genome for up to 65 species into our website. We detail the supported functionalities and workflow of our website in the following section.

### Supported indices and species

To achieve a more comprehensive evaluation of rare codon usage, 31 commonly utilized indices and 2 motif-based metrics were implemented and integrated them into our website. These indices can be categorized into four groups according to the way they process the gene expression, as outlined in Table 1. Detailed description of all these included indices is in the Supplementary material.

The first category calculates the deviation of codon usage frequency from a “uniform” distribution, which provides an informative measurement of codon usage bias without requiring prior knowledge, indicating selection's influence on gene expression levels, such as RSCU (Relative Synonymous Codon Usage) [7] and ENC (Effective Number of Codons) [8, 9]. Codon bias indices in the second category compare codon frequency between a reference set of genes and the host organism, using different methodologies to calculate similarity scores and identify coding sequences with higher gene expression based on closely resembling codons in the reference set, such as CAI (Codon Adaptation Index) [20]and FOP (Frequency of Optimal Codons) [21, 22]. Considering that codons decoded by more frequent tRNAs are used more frequently, the third type of methods are proposed based on this positive correlation between tRNA levels and codon usage, including tAI (tRNA Adaptation Index) [29] and P2 index [31]. To model the influence of longer sequences and regulatory codes on gene expression and intracellular processes, the fourth category of methods employ advanced statistical methods to analyze complex patterns of codon usage, such as GC content [32] and ENcp (Effective Number of Codon Pairs) [33].

Our website offers an extensive selection of 65 expression host species, with each reference species providing users with access to two codon tables. One of codon tables is directly cited from commonly used Codon Usage Database (https://www.kazusa.or.jp/codon/), while the other is calculated on the basis of genomic coding sequences downloaded from well-annotated CDS of NCBI Genomes FTP (https://www.ncbi.nlm.nih.gov/home/genomes/). Detailed information about supported species can be found in the Supplementary materials.

### Web server

The website interface of our Rare Codon Analysis Tool allows users to submit one or multiple sequences and choose from a comprehensive list of 65 expression host organisms. Users can submit sequences and receive analysis results in just three simple steps:

Step 1: Select your desired expression host organism along with their reference sources.

Step 2: Enter your DNA or RNA sequence(s) into the table.

Step 3: Click “Analysis” button to view detailed analysis results and attached references.

**Fig. 2:**
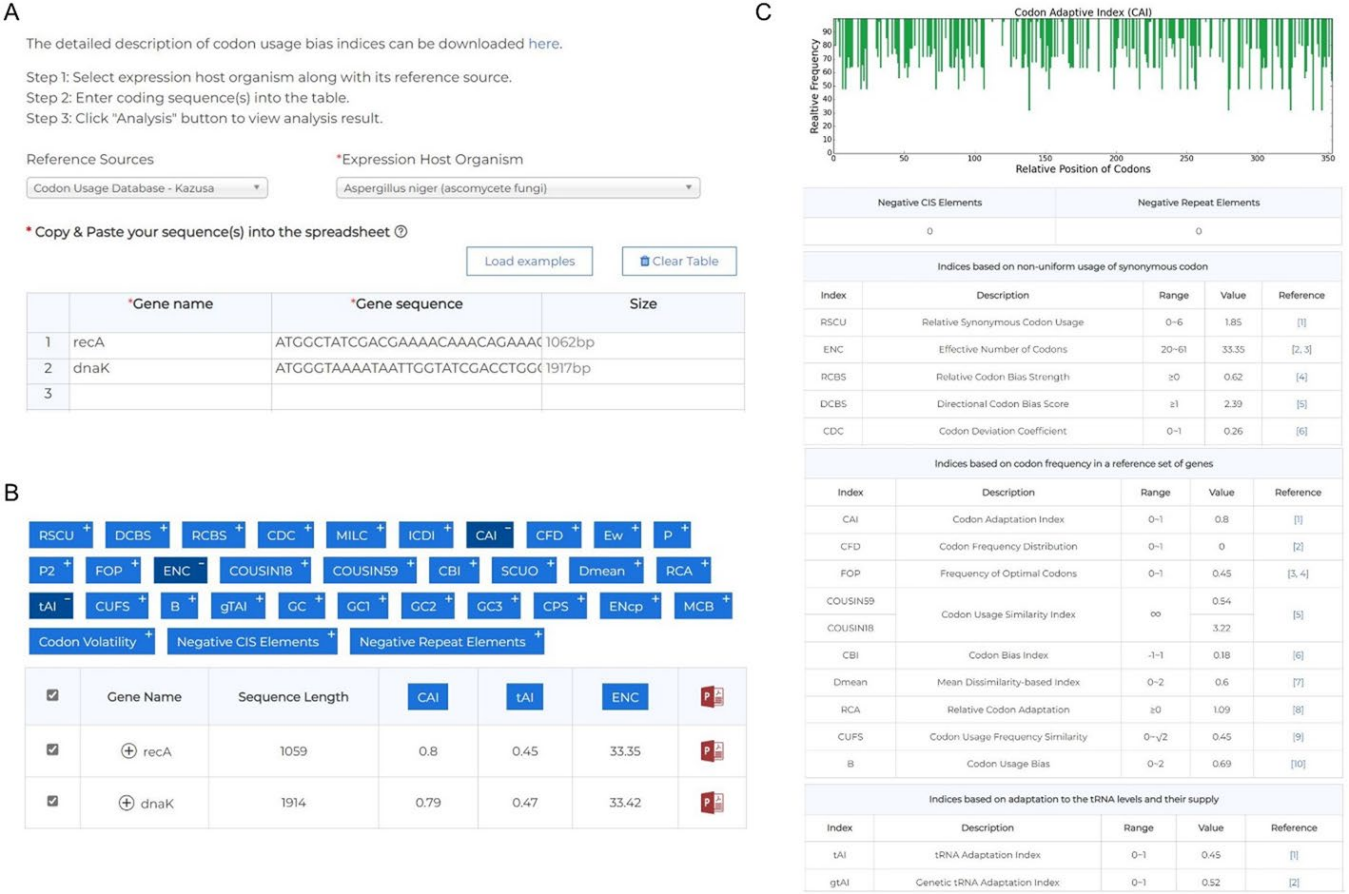
GenRCA website interface. **A** The input interface of GenRCA. Users need to choose the reference source and expression host organism, and input the queried sequences. **B** Highlighted indices of interest shown on the top. Users can customize which metrics to show on the top by clicking the corresponding button, and learn the detailed description of the index by placing the mouse over the button. **C** Main results panel. The graphical representation of CAI, two motif-based metrics and 31 CUB indices are displayed

As indicated in Fig. 2A, when multiple sequences are provided, unique gene names should be specified. You can directly copy multiple sequences from your file and paste them into the spreadsheet in our website. We implemented a “Load examples” button that, when clicked, loads two selected sequences into the spreadsheet to serve as a simple illustrative example. We restrict our analysis to sequences with lengths between 60 and 12,000 bp. Once sequences are provided, several preprocessing steps will be conducted. Firstly, we remove all symbols apart from alphabetic characters, including special symbols such as \t and \n. Additionally, any stop codons located at the end of the sequence will be eliminated. If a stop codon or concatenated bases appears in the middle, which refer to sequences containing repeated or continuous bases that do not form valid codons, it will be excluded from further analysis. Additionally, sequences whose lengths are not multiples of 3 will be removed, as they are not valid coding sequences.

Once the “Analysis” button is clicked, the comprehensive analysis results would be displayed in just a few seconds. The results on our website are presented in an interactive manner, allowing users to choose metrics of most interest to display on the top from 31 indices (Fig. 2B), serving as a quick view of the analysis. Indices shown on the top is totally customized and determined by the user. By placing the mouse over the index in the navigation bar, users can view the corresponding description and mathematical definition. In the main results panel, a graphical representation of CAI is first presented, followed by two motif-based values that have been proved to be related to gene expression: number of negative CIS elements and number of negative repeat elements. Then, detailed results and reference ranges for all 31 indices are provided below, organized into four sections based on their categories (Fig. 2C). Additionally, users can download an analysis report in PDF format for further reference.

## Results and discussion

**Fig. 3:**
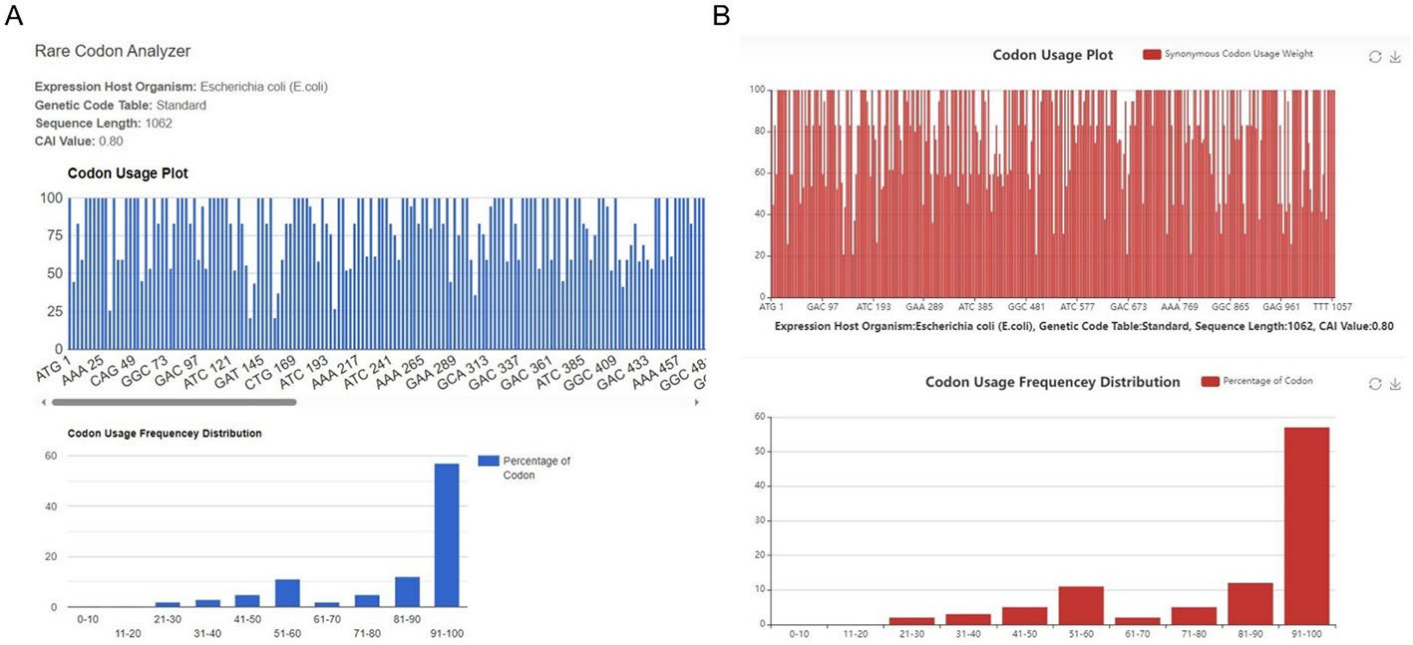
Results shown on the website of available tools. recA gene is used as an example, which is the same as in Fig. 2. **A** https://www.biologicscorp.com/tools/RareCodonAnalyzer**B** http://www.detaibio.com/tools/rare-codon-analyzer.html

The main contribution of our study is integrating a large number of CUB indices for a comprehensive rare codon analysis, compared to available tools (Table 1). As an example, we used recA gene as a query to search on two existing tools: rare codon analyzer on Biologics International Corp and Detaibio. Both tools presented only codon usage frequency distribution and codon adaptive index (CAI) score (Fig. 3), while our tool provides thirty more CUB indices for enriched information (Fig. 2C).

**Fig. 4:**
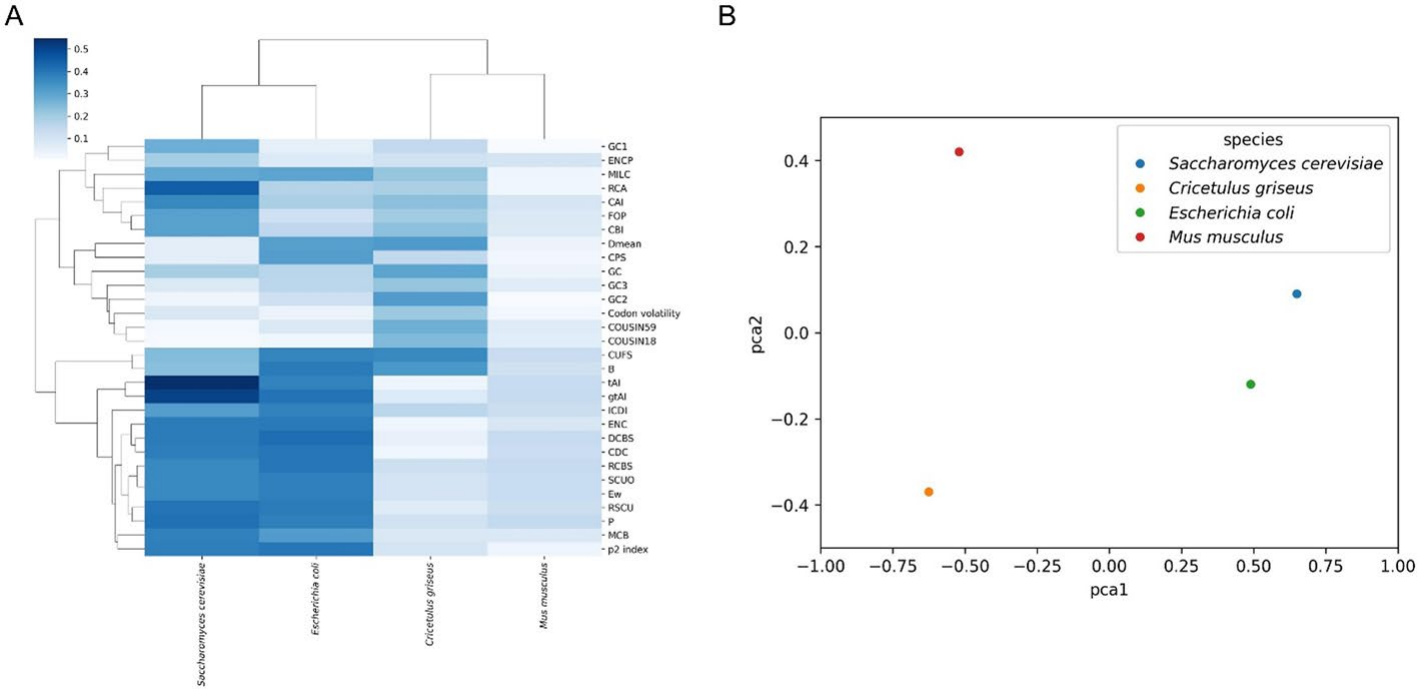
Correlation analysis between protein expression and CUB indices. **A** Heatmap of Spearman correlation coefficients between protein expression and CUB indices for four species with dendrogram. **B** PCA plot of Spearman correlation coefficients for four species

To validate the usefulness and necessity of a comprehensive rare codon analysis tool, we conducted correlation analysis between protein expression and a set of CUB indices for four species: *Saccharomyces cerevisiae* [37], *Cricetulus griseus* [38], Escherichia coli [39], and Mus musculus [40], based on Spearman correlation coefficients. We extracted protein IDs and protein expression values from the supplementary files of these four papers, and obtained corresponding DNA coding sequences by searching on the Ensembl database. Then, all indices were calculated by inputting coding sequences to our website (Supplementary data). Surprisingly, it is found that indices showing the strongest correlation with protein expression differ significantly among species, suggesting that there is no universally applicable index for all species (Fig. 4A). Notably, commonly used indices for assessing protein expression, such as CAI, did not perform as expected across these species, which is consistent with previous studies [41–43], indicating that there is no one-fits-all index for all species. Hence, our website offers up to 31 indices for researchers to comprehensively assess the protein expression levels rather than only relying on one or two commonly used indices. We also conducted a principal component analysis (PCA) on the Spearman correlation results used above, and showed a scatter plot of these four species. As shown in Fig. 4B, Saccharomyces cerevisiae and Escherichia coli, both unicellular organisms, are clustered closely, while being far away from other two multicellular organisms, indicating that the use of multiple CUB indices can reveal evolutionary processes. In the future, these comprehensive list of 31 indices can serve as input features for a machine learning model to better predict the protein expression. For example, a simple linear regression model could be trained to associate the relationship between the expression level and a set of 31 indices.

Though being the most comprehensive rare codon analysis website to date, our proposed website still has some limitations, in terms of the incompleteness of supported species and reference sources. In the future, we will integrate more types of species into our website, aiming to include as many species as possible from the Codon Usage Database and the NCBI genome database. Besides, we will regularly update reference sources information, such as synchronizing periodically with the Codon Usage Database. Moreover, we may allow users to customize species and reference sources by uploading a codon table.

## Conclusions

In this work, we made significant updates to our highly cited rare codon analysis website, which allows users to evaluate and determine whether codon optimization is necessary to enhance gene expression in the target host organism. We expanded the number of supported CUB indices to 31 and included codon tables for 65 expression host species. Additionally, we incorporated a batch processing feature, allowing users to conveniently analyze multiple sequences simultaneously, thereby improving the overall user-friendliness of the website. Considering that there is no single index suitable for every species, our website offers opportunities for researchers to comprehensively evaluate the protein expression level of coding sequences by considering all 31 supported indices together for their species of interest.

## Supplementary Information

Additional file1 (DOCX 121 KB)

Additional file2 (XLSX 4102 KB)

## Data Availability

Project name: GenRCA rare codon analysis tool. Project home page: https://www.genscript.com/tools/rare-codon-analysis. Operating system: Platform independent. Programming language: Python 3. Other requirements: Not applicable. License: Apache License 2.0. Any restrictions to use by non-academics: None. The Datasets used in this study are available in the Supplementary data file.

## Abbreviations

CUB: Codon usage bias
RSCU: Relative synonymous codon usage
ENC: Effective number of codons
CAI: Codon adaptation index
FOP: Frequency of optimal codons
tAI: TRNA adaptation index
ENcp: Effective number of codon pairs
PCA: Principal component analysis
