# Supplementary material for: GenRCA: a user-friendly rare codon analysis tool for comprehensive evaluation of codon usage preferences based on coding sequences in genomes
Source: BMC Bioinformatics. 2024 Sep 27;25:309. doi: 10.1186/s12859-024-05934-z (PMC11438159; doi:10.1186/s12859-024-05934-z)
Supplement: Supplementary file 1 — Additional file1 (DOCX 121 KB) [file 12859_2024_5934_MOESM1_ESM.docx]

**GenRCA: a user-friendly rare codon analysis tool for comprehensive evaluation of codon usage preferences based on coding sequences in genomes**

Kunjie Fan^1#^, Yuanyuan Li^2#^, Zhiwei Chen^2#^ and Long Fan^1,2^*

^1^Production and R&D Center I of LSS, GenScript (Shanghai) Biotech Co.,Ltd., Shanghai, China, ^2^Production and R&D Center I of LSS, GenScript Biotech Corporation, Nanjing, China

^#^Authors contributed equally to this study.

*To whom correspondence should be addressed.

# **Content**

[Content 2](#_Toc149831167)

[Brief Introduction 4](#_Toc149831168)

[Expression Host Species 5](#_Toc149831169)

[Commonly Used Index Descriptors 8](#_Toc149831170)

[Indices based on non-uniform usage of synonymous codon 8](#_Toc149831171)

[Relative Synonymous Codon Use (RSCU) 9](#_Toc149831172)

[Effective Number of Codons (ENC) 9](#_Toc149831173)

[Relative Codon Bias Strength (RCBS) 10](#_Toc149831174)

[Directional Codon Bias Score (DCBS) 10](#_Toc149831175)

[Codon Deviation Coefficient (CDC) 11](#_Toc149831176)

[Measure Independent of Length and Composition (MILC) 12](#_Toc149831177)

[Intrinsic Codon Deviation Index (ICDI) 12](#_Toc149831178)

[Synonymous Codon Usage Order (SCUO) 13](#_Toc149831179)

[Weighted Sum of Relative Entropy (Ew) 14](#_Toc149831180)

[Codon Preference (P) 14](#_Toc149831181)

[Maximum-likelihood Codon Bias (MCB) 15](#_Toc149831182)

[Indices based on codon frequency in a reference set of genes 15](#_Toc149831183)

[Codon Adaptation Index (CAI) 15](#_Toc149831184)

[Frequency of Optimal Codons (FOP) 16](#_Toc149831185)

[Codon Usage Similarity Index (COUSIN) 16](#_Toc149831186)

[Codon Bias Index (CBI) 17](#_Toc149831187)

[Mean Dissimilarity-based Index (Dmean) 18](#_Toc149831188)

[Relative Codon Adaptation (RCA) 19](#_Toc149831189)

[Codon Usage Frequency Similarity (CUFS) 19](#_Toc149831190)

[Codon Usage Bias (B) 20](#_Toc149831191)

[Indices based on adaptation to the tRNA levels and their supply 21](#_Toc149831192)

[tRNA Adaptation Index (tAI) 21](#_Toc149831193)

[Genetic tRNA Adaptation Index (gtAI) 22](#_Toc149831194)

[P2 Index 23](#_Toc149831195)

[Indices based on complex statistical patterns of codon usage 23](#_Toc149831196)

[GC Content at the First Position of Synonymous Codons (GC1) 23](#_Toc149831197)

[GC Content at the Second Position of Synonymous Codons (GC2) 23](#_Toc149831198)

[GC Content at the Third Position of Synonymous Codons (GC3) 24](#_Toc149831199)

[GC Content (GC) 24](#_Toc149831200)

[Effective Number of Codon Pairs (ENcp) 24](#_Toc149831201)

[Codon Pair Score (CPS) 25](#_Toc149831202)

[Codon Volatility 25](#_Toc149831203)

[Analysis 27](#_Toc149831204)

[Summary 28](#_Toc149831205)

[References 29](#_Toc149831206)

# Brief Introduction

Codon Usage Bias (CUB) indices offer a powerful and versatile tool for understanding the patterns and implications of codon usage across different organisms, without the need for extensive experimental work. Currently existing rare codon analysis websites only generate basic plots of codon usage frequency distributions without conducting further analysis. Moreover, these websites offer limited reference species which fails to encompass commonly used species, and lack the capability for batch processing of sequences and do not provide download functions.

In this work, we have made significant updates to the origin rare codon analysis website. These updates include expanding the number of codon preference indices to 31 and increasing the number of expression host organisms to 65, A detailed description of each index and its corresponding calculation process can be found in the subsequent sections. We have also introduced a new feature that enables batch processing of gene sequences, making it easier to analyze multiple sequences simultaneously. These updates aim to improve the user experience and provide more comprehensive and efficient analysis capabilities. The updated website can still be accessed using the original URL: [Rare Codon Analysis Tool (genscript.com)](https://www.genscript.com/tools/rare-codon-analysis).

# Expression Host Species

On our website, users can explore a wide range of 65 expression host species, each of which offers access to two codon tables. The first codon table is generated based on the counting of reference sequences, while the second one can be downloaded from the Codon Usage Database at <https://www.kazusa.or.jp/codon/>.

The reference sequences of *Nicotiana benthamiana* and *Saccharum spontaneum* on our website are sourced from https://nbenthamiana.jp/downloads and https://ftp.ensemblgenomes.ebi.ac.uk/pub/plants/release-56/fasta/saccharum_spontaneum/, respectively. Additionally, the genomes of the other 63 species are obtained from NCBI (https://www.ncbi.nlm.nih.gov/), and details of these expression host species can be founded in **Supplementary Table 1**.

**Supplementary Table 1.** Taxonomy of 65 species and sources of genomes on NCBI

| **Scientific Name** | **Taxon** | **Reference Sequence** |
| --- | --- | --- |
| *Arabidopsis thaliana (thale cress)* | 3702 | GCF_000001735.4 |
| *Aspergillus niger (ascomycete fungi)* | 5061 | GCF_000002855.3 |
| *Aspergillus oryzae (ascomycete fungi)* | 5062 | GCF_000184455.2 |
| *Bacillus subtilis (firmicutes)* | 1423 | GCF_000009045.1 |
| *Bombyx mori (domestic silkworm)* | 7091 | GCF_014905235.1 |
| *Bos taurus (cattle)* | 9913 | GCF_002263795.2 |
| *Brassica napus (rapeseeds)* | 3708 | GCF_020379485.1 |
| *Caenorhabditis elegans (nematodes)* | 6239 | GCF_000002985.6 |
| *Canis lupus familiaris (dog)* | 9615 | GCF_011100685.1 |
| *Caulobacter crescentus (a-proteobacteria)* | 155892 | GCF_000022005.1 |
| *Rhodobacter sphaeroides (a-proteobacteria)* | 1063 | GCF_000021005.1 |
| *Cervus elaphus (red deer)* | 9860 | GCF_910594005.1 |
| *Chlamydomonas reinhardtii (green algae)* | 3055 | GCF_000002595.2 |
| *Clostridium acetobutylicum (firmicutes)* | 1488 | GCF_000218855.1 |
| *Corynebacterium glutamicum (high G+C Gram-positive bacteria)* | 1718 | GCF_000404185.1 |
| *Cricetulus griseus (Chinese hamster)* | 10029 | GCF_000223135.1 |
| *Cyberlindnera jadinii (budding yeasts)* | 4903 | GCF_001661405.1 |
| *Danio rerio (zebrafish)* | 7955 | GCF_000002035.6 |
| *Drosophila melanogaster (fruit fly)* | 7227 | GCF_000001215.4 |
| *Escherichia coli (E. coli)* | 386585 | GCF_000008865.2 |
| *Gallus gallus (chicken)* | 9031 | GCF_016699485.2 |
| *Glycine max (soybean)* | 3847 | GCF_000004515.6 |
| *Gossypium hirsutum (cotton)* | 3635 | GCF_007990345.1 |
| *Homo sapiens (human)* | 9606 | GCF_000001405.40 |
| *Hordeum vulgare (barley)* | 4513 | GCF_904849725.1 |
| *Kluyveromyces lactis (budding yeasts)* | 28985 | GCF_000002515.2 |
| *Pichia pastoris (budding yeasts)* | 4922 | GCA_001708105.1 |
| *Lactobacillus casei (firmicutes)* | 1582 | GCF_000829055.1 |
| *Lactococcus lactis (firmicutes)* | 1358 | GCF_020463755.1 |
| *Leishmania tarentolae (kinetoplastids)* | 5689 | GCA_009731335.1 |
| *Macaca mulatta (Rhesus monkey)* | 9544 | GCF_003339765.1 |
| *Mesocricetus auratus (golden hamster)* | 10036 | GCF_017639785.1 |
| *Mus musculus (house mouse)* | 10090 | GCF_000001635.27 |
| *Mycobacterium smegmatis (high G+C Gram-positive bacteria)* | 1772 | GCF_013349145.1 |
| *Nicotiana benthamiana (eudicots)* | 4100 | / |
| *Nicotiana tabacum (common tobacco)* | 4097 | GCF_000715135.1 |
| *Pichia angusta (budding yeasts)* | 4905 | GCF_019207475.1 |
| *Ogataea polymorpha (budding yeasts)* | 460523 | GCF_001664045.1 |
| *Oleidesulfovibrio alaskensis G20 (bacteria)* | 207559 | GCF_000012665.1 |
| *Oryctolagus cuniculus (rabbit)* | 9986 | GCF_009806435.1 |
| *Oryza sativa Indica (long-grained rice)* | 39946 | GCA_000004655.2 |
| *Oryza sativa Japonica Group (Japanese rice)* | 39947 | GCF_001433935.1 |
| *Paraliobacillus quinghaiensis (firmicutes)* | 470815 | GCF_003426025.1 |
| *Phaseolus vulgaris (eudicots)* | 3885 | GCF_000499845.1 |
| *Plasmodium falciparum (malaria parasite P. falciparum)* | 36329 | GCF_000002765.5 |
| *Populus trichocarpa (black cottonwood)* | 3694 | GCF_000002775.5 |
| *Pseudomonas putida (g-proteobacteria)* | 303 | GCF_000412675.1 |
| *Rattus norvegicus (Norway rat)* | 10116 | GCF_015227675.2 |
| *Rhodococcus opacus (high G+C Gram-positive bacteria)* | 37919 | GCF_020542785.1 |
| *Saccharomyces cerevisiae (baker's yeast)* | 4932 | GCF_000146045.2 |
| *Saccharum spontaneum (fodder cane)* | 62335 | / |
| *Solanum lycopersicum (tomato)* | 4081 | GCF_000188115.5 |
| *Spodoptera frugiperda (fall armyworm)* | 7108 | GCF_023101765.2 |
| *Staphylococcus carnosus (firmicutes)* | 1281 | GCF_900458435.1 |
| *Streptomyces coelicolor (high G+C Gram-positive bacteria)* | 1902 | GCF_013317105.1 |
| *Streptomyces lividans (high G+C Gram-positive bacteria)* | 1916 | GCF_000739105.1 |
| *Sus scrofa (pig)* | 9823 | GCF_000003025.6 |
| *Synechococcus elongatus (cyanobacteria)* | 32046 | GCF_000012525.1 |
| *Thermothelomyces heterothallica (ascomycete fungi)* | 1920207 | GCF_000226095.1 |
| *Trichoderma reesein (ascomycete fungi)* | 51453 | GCF_000167675.1 |
| *Trichoplusia ni (cabbage looper)* | 7111 | GCF_003590095.1 |
| *Triticum aestivum (bread wheat)* | 4565 | GCF_018294505.1 |
| *Xenopus laevis (African clawed frog)* | 8355 | GCF_017654675.1 |
| *Yarrowia lipolytica (budding yeasts)* | 4952 | GCF_000002525.2 |
| *Zea mays (maize)* | 4577 | GCF_902167145.1 |

The taxon is the distinctive identifier for the curated classification and nomenclature of all organisms within the National Center for Biotechnology Information (NCBI). In cases where a particular species lacks RefSeq data within the NCBI database, we will resort to utilizing the Genebank data instead.

To ensure accuracy, we performed several modifications on these reference sequences: (1) Sequences related to nematoplasts, chloroplasts, plastids, and parietal plasmodesmata were discarded. (2) Repetitive sequences were eliminated. (3) Sequences with base numbers that are not multiples of three were removed. (4) Sequences containing concatenated bases were deleted. After these rigorous processes, the occurrence frequency of 64 codons in the genomes of the various species was counted.

The tRNA copy numbers for each species were obtained from Genomic tRNA Database (http://gtrnadb.ucsc.edu/). However, we were unable to calculate tAI and gtAI for 17 species for which data was not available in GtRNAdb: *Trichoplusia ni (High Five Cells), Hordeum vulgare, Leishmania tarentolae, Oryza sativa Indica, Pichia angusta, Streptomyces coelicolor, Xenopus laevis (African clawed frog), Gossypium hirsutum, Nicotiana benthamiana, Saccharum spontaneum, Paraliobacillus quinghaiensis, Ogataea polymorpha, Mesocricetus auratus, Phaseolus vulgaris, Thermothelomyces heterothallica, Cervus elaphus*, and *Oleidesulfovibrio alaskensis*. As a result, the calculations of these species for tAI and gtAI are not currently available on our website.

# Commonly Used Index Descriptors

To enhance the comprehensiveness of our evaluation of rare codon usage, we have categorized these indices on our website into 4 major categories based on the gene expression process. These categories allow for a systematic analysis of the various aspects of codon usage biases. A detailed description of each index and its corresponding calculation process can be found in the subsequent sections, providing a comprehensive resource for researchers in the field.

## Indices based on non-uniform usage of synonymous codon

Indices in this section calculate the deviation of codon usage frequency from a "uniform" or expected "background" distribution. An increased CUB observed in a certain gene or genomic region suggests that selection has acted upon it, favoring certain codons that impact expression levels. Generally, these indices demonstrate a consistent relationship with the level of codon usage uniformity or frequency. At the extremes, these methods will identify cases where only one codon is used (maximal bias) or where all codons are utilized with equal frequency (minimum bias). It is worth noting that these indices are based solely on the coding sequence, thus offering an informative measurement of CUB without the need for prior knowledge.

### Relative Synonymous Codon Use (RSCU)

The Relative Synonymous Codon Usage (RSCU)^1^ value for a codon represents the ratio between the observed frequency of a particular codon and the expected frequency, assuming equal usage of all synonymous codons for the corresponding amino acid. The formula for calculating RSCU is as follows:

$${RSCU}_{ij} = \frac{x_{ij}}{\frac{1}{n_{i}}\sum_{j=1}^{n_{i}} x_{ij}}$$

where $x_{ij}$ denotes the number of occurrences of the $j$-th codon for the $i$-th amino acid, while $n_{i}$ represents the degeneracy for the $i$-th amino acid. It is worth noting that if the codon is missing from the gene, we consider the RSCU value of this codon as 0.0001. The RSCU value of 1 indicates the average synonymous codon usage. A codon with an RSCU value greater than 1 is considered to be preferred or overrepresented, as it is used more frequently than expected. Conversely, a codon with an RSCU value less than 1 is considered to be unpreferred or underrepresented, as it is used less frequently than expected based on the assumption of equal usage of all synonymous codons for the corresponding amino acid. Therefore, the RSCU value is a useful tool for identifying codon bias and for gaining insight into the regulation of gene expression. Furthermore, the RSCU values also serve as weights for codons in various indices that require normalization of codon count into codon frequency and elimination of dependency on gene length, such as Codon Adaptation Index (CAI), Intrinsic Codon Deviation Index (ICDI), etc.

The RSCU of a gene on our website can be computed by taking the arithmetic mean of all codons. The calculation can be performed as follows:

$$RSCU\left( g \right)=\frac{\sum_{k=1}^{L} {rscu}_{k}}{L}$$

where $L$ is the length of a gene measured in codons and ${rscu}_{k}$ is the RSCU value for the $k$-th codon in the gene.

### Effective Number of Codons (ENC)

The mathematical formula utilized in the calculation of the Effective Number of Codons (ENC)^2,3^ is based on principles derived from the field of population genetics. ENC takes into account the degeneracy level of amino acids and computes the total number of distinct codons used in a sequence. This index can assess the extent of bias or preference in the usage of synonymous codons within a particular gene or genome.

The theoretical value of ENC ranges from 61 (when there is no bias and all synonymous codons are used uniformly) to 20 (when there is maximal bias or preference observed in synonymous codon usage). A lower ENC value indicates a stronger inclination towards specific codons for a particular amino acid, while a higher ENC value suggests a more balanced or uniform utilization of synonymous codons.

For an amino acid (AA) with a degeneracy of $k$, meaning it has $k$ synonymous codons, each with counts $n_{1}$, $n_{2}$, ..., $n_{k}$, $n=\sum_{i=1}^{k} n_{i}$ and $p_{i}=n_{i}/n$, the effective number of codons for each amino acid ($\hat{N}_{cAA}$) is calculate as follows:

$$\hat{N}_{cAA}=\frac{1}{F_{AA}}$$

where

$$F_{AA}=\frac{n\sum_{i=1}^{k} {p_{i}}^{2}-1}{\left( n-1 \right)} n>1$$

It is important to mention that if there is a maximum bias in the usage of codons for an amino acid, meaning that only one synonymous codon is used, the $F_{AA}$ for that amino acid is 1.

Finally, for the standard genetic code, the formula for calculating ENC ($\hat{N}_{c}$) for a gene is as follows:

$$\hat{N}_{c}=2+\frac{9}{\bar{F}_{2}}+\frac{1}{F_{3}}+\frac{5}{\bar{F}_{4}}+\frac{3}{\bar{F}_{6}}$$

where $\bar{F}_{i}$ ($i$ = 2, 3, 4 and 6) represents the average values of $F_{AA}$ for all amino acids with degeneracy $i$. It is worth noting that rarely used amino acids (indicated by a $F_{AA}$ value of 0) should be regarded as absent and are not taken into consideration when calculating the averages. However, if isoleucine (Ile) is absent or rarely used ($F_{3}$ = 0), the value of $F_{3}$ should be calculated as the average of $\bar{F}_{2}$ and $\bar{F}_{4}$.

### Relative Codon Bias Strength (RCBS)

The Relative Codon Bias (RCB)^4^ is a metric used to assess the level of bias in codon usage. It is calculated by comparing the observed frequency of a specific codon to the expected frequency assuming random codon usage, taking into account the biased base composition at three sites as observed in the sequence being studied. This difference is then divided by the expected frequency, providing a normalized measure of codon usage bias that is independent of overall base composition variability. The RCB of the codon $xyz$ is calculated as follows:

$$d_{xyz}=\frac{f\left( x,y,z \right)-f_{1}\left( x \right)\cdot f_{2}\left( y \right)\cdot f_{3}\left( z \right)}{f_{1}\left( x \right)\cdot f_{2}\left( y \right)\cdot f_{3}\left( z \right)}$$

Specifically, $f\left( x,y,z \right)$ represents the observed frequency of codon $xyz$, where $x$, $y$, and $z$ denote the first, second, and third nucleotides of that codon, respectively. Additionally, $f_{1}\left( x \right)$, $f_{2}\left( y \right)$ and $f_{3}\left( z \right)$ represent the observed frequencies of the individual bases $x$, $y$, and $z$ at positions 1, 2, and 3 of the codons. Rare codons are assigned lower $d_{xyz}$ values, typically approaching -1. Conversely, highly frequent codons are assigned higher $d_{xyz}$ values, which can reach a value of 1.

The RCBS (Relative Codon Bias Strength)^4^ is designed to consider base compositional bias, enabling a more robust estimation of highly favored codon frequencies while accounting for other characteristics of the coding sequence, including GC content bias. It provides a quantitative evaluation of the deviation from expected codon frequencies, taking into account the underlying RCB factors observed in the gene's sequence. To calculate the RCBS for a gene with $L$ codons, the following formula is used:

$$RCBS=\left( \prod_{i=1}^{L} \left( 1+d_{xyz}^{i} \right) \right)^{1/L} -1=\exp\frac{1}{L}\sum_{i=1}^{L} \left( 1+d_{xyz}^{i} \right)-1$$

A value of RCBS close to 0 suggests a lack of bias in codon usage. Conversely, a value greater than 0.5 indicates a favorable preference for specific codon usage, which signifies high gene expression.

### Directional Codon Bias Score (DCBS)

According to the RCBS (Relative Codon Bias Strength)^4^ formula, rare codons have negative RCB values, approaching -1, while very frequent codons have positive RCB values, approaching 1. As a result, the presence of very rare codons decreases the final RCBS score of a gene, while the presence of very frequent codons increases it. However, genes with a high Codon Usage Bias (CUB) should include both very frequent and very rare codons. To ensure that both positive and negative codon usage biases contribute to the same direction and increase the RCBS score, a modified version called The Directional Codon Bias Score (DCBS)^5^ has been proposed.

DCBS considers both overrepresented and underrepresented codons, providing a more accurate evaluation of codon bias in a gene by considering the directionality of codon usage bias. To calculate DCBS, the Directional Codon Bias (DCB) of a codon triplet $xyz$ is defined as follows:

$$d_{xyz}=max\left( \frac{f\left( x,y,z \right)}{f_{1}\left( x \right)\cdot f_{2}\left( y \right)\cdot f_{3}\left( z \right)},\frac{f_{1}\left( x \right)\cdot f_{2}\left( y \right)\cdot f_{3}\left( z \right)}{f\left( x,y,z \right)} \right)$$

Identical to RCBS, $f\left( x,y,z \right)$ represents the observed frequency of codon $xyz$, and $f_{1}\left( x \right)$, $f_{2}\left( y \right)$ and $f_{3}\left( z \right)$ represent the observed frequencies of the individual bases $x$, $y$, and $z$ at positions 1, 2, and 3 of the codons.

The DCBS of a gene, which is composed of $L$ codons, is calculated as follows:

$$DCBS=\frac{\sum_{i=1}^{L} d_{xyz}}{L}$$

Similar to RCBS, a value close to 0 for DCBS indicates a lower degree of codon preference, while a higher value indicates a stronger bias in codon usage.

### Codon Deviation Coefficient (CDC)

The Codon Deviation Coefficient (CDC)^6^ is based on the cosine distance metric between the expected and the observed codon usage. This metric takes into account background nucleotide compositions (BNC) specific to codon positions, thereby allowing for a more precise evaluation of CUB in diverse genetic sequences.

In this context, the four nucleotides (adenine, thymine, guanine, and cytosine) are denoted as A, T, G, and C, while the GC content and purine content are represented as S and R, respectively. The expected nucleotide contents (A, T, G, C) at codon position $i$ ($i$ = 1, 2, 3) is derived based on the observed positional GC and purine contents, which can be formulated as follows:

$$A_{i}=\left( 1-S_{i} \right)R_{i}$$

$$T_{i}=(1-S_{i}){(1-R}_{i})$$

$$G_{i}=S_{i}R_{i}$$

$$C_{i}=S_{i}\left( 1-R_{i} \right)$$

For any sense codon $xyz\left( x,y,z\in A,T,G,C \right)$, the expected usage $\pi xyz$ is defined as the product of its constituent expected nucleotide contents $x_{1}y_{2}z_{3}$, normalized by the sum over all sense codons:

$$\pi xyz=\frac{x_{1}y_{2}z_{3}}{\sum_{abc} w_{abc}a_{1}b_{2}c_{3}}$$

where

$$w_{abc}=\left\{ \begin{aligned} 1, if abc is a sense codon \\ 0, otherwise \end{aligned} \right.anda,b,c\in A,T,G,C$$

Also, the observed usage of the sense codon $xyz$ is normalized by the length of gene:

$$\hat{\pi}xyz=\frac{F_{xyz}}{L}$$

where $F_{xyz}$ represent the observed frequency of that codon in the gene, and $L$ is the length of a gene measured in codons.

Then, CDC is calculated using the cosine distance metric, which measures the similarity between the expected ($\pi$) and observed ($\hat{\pi}$) codon usage patterns:

$$CDC=1-\frac{\sum_{xyz} \pi xyz\times\hat{\pi}xyz}{\sqrt{\sum_{xyz} {\pi xyz}^{2}\times\sum_{xyz} {\hat{\pi}xyz}^{2}}}$$

The CDC ranges from 0 to 1, where a value closer to 0 indicates a closer correspondence between the expected and observed codon usage patterns, suggesting a lower level of bias in codon usage. Conversely, a CDC value closer to 1 represents the greater level of codon usage bias.

### Measure Independent of Length and Composition (MILC)

The Measure Independent of Length and Composition (MILC)^7^ quantifies the variation in codon usage by calculating a log-likelihood ratio between the expected and observed counts of codons. MILC is a versatile measure that remains unaffected by alterations in gene length and overall nucleotide composition, and introducing little noise into measurements. This approach yields similar numerical results to the widely used $\chi^{2}$ test, but may offer theoretical advantages in statistical analyses. Only sequences consisting of 80 codons or more are recommended to be analyzed using MILC. A higher value indicates a stronger bias in codon usage, which signifies higher gene expression.

The individual contribution ($M_{a}$) of each amino acid $a$ to the MILC statistic is calculated as follows:

$$M_{a}=\sum_{c\in a} O_{c}In\frac{O_{c}}{E_{c}}=\sum_{c\in a} O_{c}In\frac{f_{c}}{g_{c}}$$

where $O_{c}$ represents the observed count of codon $c$ in a gene, $E_{c}$ denotes the expected count of the same codon, $f_{c}$ signifies the frequency of codon $c$ in a gene, and $g_{c}$ represents the expected frequency of the same codon. It is important to note that the sum of $f_{c}$ or $g_{c}$ over all codons for each amino acid should equal 1. In addition, the $O_{c}/E_{c}$ ratio is mathematically equal to, and can be replaced by $f_{c}/g_{c}$.

Then, MILC can be calculated by the following formula:

$$MILC=\frac{\sum_{a} M_{a}}{L}-C$$

where $L$ represents the gene length measured in codons, aiming to account for the expected increase associated with the total number of codons. The correction factor $C$ is employed to address the issue of overestimating the overall bias in shorter sequences. In the case where the codon usage in the gene aligns with the expected distribution and all amino acids are present, the occurrence of sampling errors leads to an increase in the $\chi^{2}$ score by 41, and an increase in the ‘scaled’ $\chi^{2}$ by $41/L$. Consequently, the correction factor C can be computed using the following formula:

$$C=\frac{\sum_{a} \left( r_{a}-1 \right)}{L}+0.5$$

where $r_{a}$ represents the number of possible codons for the amino acid $a$, which corresponds to its degeneracy class. Only the amino acids that are actually present at least once in the sequence contribute to the calculation of $C$. For example, if a gene lacks one of the amino acids with a four-fold degeneracy, $C$ would be calculated as 38/$L$. In cases where the observed frequencies closely match the expected codon distribution, MILC values may become negative. To compensate for this, a constant of 0.5 is added to the correction factor $C$.

### Intrinsic Codon Deviation Index (ICDI)

The Intrinsic Codon Deviation Index (ICDI)^8^ utilizes the RSCU^1^ and the degeneracy of amino acids in the sequence, giving equal weight to all included amino acids. It can be applied to evaluate codon bias in genes from species without knowledge of optimal codons, and has the potential to help predict gene functionality. A gene with strong bias, using only one codon per amino acid, would have an ICDI value of 1, while a gene utilizing all codons equally would have avalue of 0.

The ICDI was calculated through the following steps. Firstly, for each of the 18 amino acids (methionine and tryptophan excluded) with $k$ number of synonymous codons, the value of $S_{k}$ is defined as follows:

$$S_{k}=\sum\frac{\left( {rscu}_{i}-1 \right)^{2}}{k\left( k-1 \right)}$$

where ${rscu}_{i}$ is the RSCU^1^ value of the $i$-th codon. It is worth noting that if the amino acid is absent in the gene sequence, the $S$ value for that particular amino acid is considered as 0.0001. For the standard genetic code, the value of $k$ corresponds to the degeneracy of the codons, which can be 2, 3, 4, or 6 triplets encoding for the same amino acid. Then the ICDI value for a gene is calculated as follows:

$$ICDI=\frac{\sum S_{2}+S_{3}+\sum S_{4}+\sum S_{6}}{18}$$

### Synonymous Codon Usage Order (SCUO)

The Synonymous Codon Usage Order (SCUO)^9,10^ is based on an informatics method, using Shannon informational theory and maximum entropy theory to provide an estimate for the orderliness of synonymous codon usage. Entropy is a measure of uncertainty or information content in a probabilistic system. Shannon entropy quantifies the average amount of information needed to describe or predict the outcomes of a random variable. In the context of information theory, entropy represents the amount of "surprise" or "new information" associated with the occurrence of an event.

According to the information theory, the uncertainty in synonymous codon usage of the $i$-th amino acid can be quantified using a function known as the 'entropy' of the probability distribution:

$$H_{i}=-\sum_{j=1}^{k_{i}} p_{ij}logp_{ij}$$

where $H_{i}$ represents the entropy of the $i$-th amino acid and $k_{i}$ is its degree of codon degeneracy. Moreover, $p_{ij}$ is the normalized frequency of $j$-th codon usage for the $i$-th amino acid, which can be calculated as follows:

$$p_{ij}=\frac{x_{ij}}{\sum_{j=1}^{n_{i}} x_{ij}}$$

It is noteworthy the case that the synonymous codons are chosen at random, without any bias or preference, each codon has an equal probability of occurrence for representing the $i$-th amino acid. This results in a uniform distribution of synonymous codons, maximizing the entropy associated with the $i$-th amino acid in each sequence. Consequently, the maximum entropy for the $i$-th amino acid within each sequence is as follows:

$$H_{i}^{max}=-log\frac{1}{k_{i}}=logk_{i}$$

Similarly, if only one of the synonymous codons is exclusively utilized for representing the $i$-th amino acid, it indicates an extreme bias in the codon usage. In this case, the entropy associated with the $i$-th amino acid in each sequence reaches the minimum value of 0.

The SUCO ($O_{i}$) can be regarded as a quantitative measure of the bias in synonymous codon usage for the $i$-th amino acid within each sequence, which is defined as the normalized difference between the maximum entropy and the observed entropy for the $i$-th amino acid in each sequence:

$$O_{i}=\frac{H_{i}^{max}-H_{i}}{H_{i}^{max}}=1-\frac{H_{i}}{H_{i}^{max}}$$

Clearly, the value of $O_{i}$ falls within the range of 0 to 1, where a value of 0 indicates that the synonymous codon usage for the $i$-th amino acid is completely random. Conversely, a value of 1 suggests an extreme bias in the synonymous codon usage. Therefore, $O_{i}$ can be interpreted as a measure of the degree of bias in the synonymous codon usage for the $i$-th amino acid within each sequence.

The weighted sum of SCUO ($O_{w}$) each sequence can be represented as:

$$O_{w}=\sum_{i=1}^{n} w_{i}O_{i}$$

where $n$ is the number of distinct amino acids (methionine and tryptophan excluded) in the sequence and $w_{i}$ represents the relative abundance or composition ratio of the $i$-th amino acid in each sequence:

$$w_{i}=\frac{\sum_{j=1}^{k_{i}} x_{ij}}{\sum_{i=1}^{n} \sum_{j=1}^{k_{i}} x_{ij}}$$

Obviously, the value of $O_{w}$ also ranges from 0 to 1. A value of 1 indicates maximum bias, while a value of 0 signifies no bias.

### Weighted Sum of Relative Entropy (Ew)

Similar to Synonymous Codon Usage Order (SCUO)^9,10^, the Weighted Sum of Relative Entropy ($E_{w}$)^11^ is is also based on Shannon's information theory and maximum entropy theory to evaluate the synonymous codon usage bias. $E_{w}$ is defined as the weighted sum of Relative Entropy ($E_{i}$):

$$E_{w}=\sum_{i=1}^{n} w_{i}E_{i}$$

where *n* is the number of distinct amino acids in the sequence and the formula for calculating $w_{i}$ for each amino acid is provided in SUCO. Furthermore, the Relative Entropy^31^ for the $i$-th amino acid ($E_{i}$) represents the evenness component of uncertainty, quantifying the ratio of the observed uncertainty in amino acid usage to the maximum possible uncertainty. This measure is calculated using the following function:

$$E_{i}=\frac{H_{i}}{H_{i}^{max}}=\frac{H_{i}}{logk_{i}}$$

It is worth noting that if degeneracy for amino acids is equal to 1, which means that the amino acids are encoded by only one codon, the denominator becomes zero, leading to an undefined value for $E_{i}$. Due to such nondegenerate amino acids cannot exhibit synonymous codon usage bias, they are typically excluded from consideration.

Clearly, the $E_{w}$ value range from 0 to 1. In contrast to $O_{w}$, a value of 0 indicates maximum bias, while a value of 1 signifies no bias.

### Codon Preference (P)

The Codon Preference (P)^12^ assesses the propensity of a specific set of codons to align with a predefined preferred usage. P is computed for all three reading frames, making it valuable for gene identification in sequenced DNA, predicting the relative expression level of genes, and detecting DNA sequencing errors that may lead to base insertions or deletions within coding sequences. A higher P value indicates a more frequent usage of preferred codons.

The preference parameter, for a codon $xyz$ is calculated as follows:

$$P_{xyz}=\frac{f_{xyz}/F_{xyz}}{r_{xyz}/R_{xyz}}$$

where $f_{xyz}$ represents the observed frequency of codon $xyz$. $F_{xyz}$ is the frequency of all the codons for an amino acid and can be calculated as follows:

$$F_{xyz}=\sum_{xyz\in a} f_{xyz}$$

Then, $r_{xyz}$ represents the frequency of codon in a random sequence, and $R_{xyz}$ is the sum of $r_{xyz}$ within the synonymous family which includes codon $xyz$. The formulae for their calculation are as follows:

$$R_{xyz}=\sum_{xyz\in a} r_{xyz}$$

$$r_{abc}=\frac{N_{x}N_{y}N_{z}}{N^{3}}$$

where $N_{x}$, $N_{y}$ and $N_{z}$ represent the observed frequencies of the individual bases $x$, $y$, and $z$ at positions 1, 2, and 3 of the codon. $N$ is the total number of bases in the sequence.

The preference of a gene $P$ is defined as the geometric mean of $P_{xyz}$:

$$P=\left（ \prod_{K=1}^{L} P_{k} \right）^{\frac{1}{L}}=\exp( \frac{1}{L}\sum_{k=1}^{L} {InP}_{k})$$

where $L$ is the length of a gene measured in codons and $P_{k}$ is the perference for the $k$-th codon in the gene.

### Maximum-likelihood Codon Bias (MCB)

The Maximum-likelihood Codon Bias (MCB)^13^ is designed to account for background nucleotide composition and can be further adapted to correct for di-nucleotide biases. This method proves valuable for estimating ancestral codon usage bias and conducting genetic population analysis. A higher value of MCB indicates a stronger bias in codon usage. It estimates the bias in codon usage by assigning weights to each amino acid. These weights are derived from the likelihood of occurrence of each amino acid, considering its frequency and codon degeneracy. Nevertheless, MCB is not a maximum-likelihood method in the strictest sense.

The calculation can be expressed as follows:

$$B_{g}=\sum\frac{B_{a}\cdot logN_{a}}{N_{a}}$$

where $N_{a}$ is the observed frequency of amino acid $a$. The bias for an individual amino acid, $B_{a}$, can be obtained using the following formula:

$$B_{a}=\sum_{c\in a} \frac{\left( O_{c}-E_{c} \right)^{2}}{E_{c}}$$

Where $O_{c}$ represents the observed count of codon $c$ in a gene, $E_{c}$ denotes the expected count of the same codon.

## Indices based on codon frequency in a reference set of genes

Indices in this section rely on the comparison to the codon frequency in a reference set of genes. These indices necessitate knowledge of the reference genome of the host organism and compare the variation in codon usage between the reference genes and the host organism. Coding sequences containing codons that closely resemble those in the reference set will receive a higher score, indicating higher gene expression. The differences among the indices lie in the methodology employed to compute the similarity score.

### Codon Adaptation Index (CAI)

The Codon Adaptation Index (CAI)^14^ serves as a measure to quantify the degree of codon adaptation within a gene by comparing its codon usage with the preferred or optimal codons, which ranges from 0 to 1. A higher CAI score suggests that the codon usage in the gene is more adapted towards the preferred codons observed in highly expressed a reference set, and a value of 1 is considered ideal. Genes with higher scores are expected to exhibit greater translation efficiency and higher protein expression levels.

To calculate CAI, the first step is to construct a reference table of RSCU values specifically for highly expressed genes in the organism under investigation. The relative adaptiveness ($w_{ij}$) of a codon is calculated as the ratio of the frequency of use of that codon to the frequency of the optimal codon for the same amino acid:

$$w_{ij}=\frac{{RSCU}_{ij}}{{RSCU}_{imax}}=\frac{x_{ij}}{x_{imax}}$$

where ${RSCU}_{imax}$ refers to the RSCU value of the codon that is most frequently used among all synonymous codons for the $i$-th amino acid, while $x_{imax}$ represents the count or frequency of occurrences of that codon.

The CAI for a gene is then calculated as the geometric mean of the $w_{k}$ values corresponding to each of the codons used in that gene:

$$CAI=\mathrm{ex}p \left( \frac{1}{L}\sum_{k=1}^{L} lnw_{k} \right)$$

where $L$ is the length of a gene measured in codons and $w_{k}$ is the w value for the k-th codon in the gene. $L$ does not have an inherent impact on CAI. However, it is worth noting that CAI values for shorter genes may exhibit increased variability due to sampling effects.

### Frequency of Optimal Codons (FOP)

The Frequency of Optimal Codons (FOP)^15,16^ is a simplified version of CAI that provides a lower-resolution assessment of codon usage bias, which is derived by calculating the ratio of the number of optimal codons (ATG, TGG and stop codons excluded) to the total number of codons present in the gene:

$$FOP=\frac{\# optimal codons in sequence}{\# total codons in sequence}$$

The value of FOP ranges from 0 to 1, representing the occurrence of the optimal codon within a gene sequence. A value of 0 indicates that the optimal codons never appear, while a value of 1 indicates that the optimal codons always appear in the gene sequence. It is noteworthy that the optimal codons can be defined based on several factors, including nucleotide chemistry, codon usage bias, and tRNA availability. In our work, optimal codons were chosen to be the codons with the highest frequency of occurrence for each amino acid in reference.

### Codon Usage Similarity Index (COUSIN)

The Codon Usage Similarity Index (COUSIN)^17^ compares the CUB of a query against that of a reference and normalizes the output using a Null Hypothesis of random codon usage. This index is valuable in identifying differential heterogeneity both between and within genomic data sets. A COUSIN score of 1 indicates that the codon usage preferences in the query are similar to those in the reference dataset. A score of 0 suggests that the codon usage preferences in the query are similar to those in the null hypothesis, where there is an equal usage of synonymous codons. Scores between 0 and 1 imply that the codon usage preferences in the query are similar to those in the reference, but with a smaller magnitude. Scores above 1 indicate that the codon usage preferences in the query are similar to those in the reference, but with a larger magnitude.

To calculate COUSIN, it is necessary to first calculate the deviation scores ${dev}_{c,a}$:

$${dev}_{c,a}=f_{c,a}^{ref}-f_{c,a}^{H_{0}}$$

where $f_{c,a}^{ref}$ is the frequency of the codon $c$ among its synonymous codons in the reference, and $f_{c,a}^{H_{0}}$ is the observed frequency of that codon among its synonymous codons in the query sequence.

Then, the weight for each codon in the reference and query gene is defined by multiplying the codon frequency in the reference by its deviation score:

$$W_{c,a}^{ref}=f_{c,a}^{ref}\times{dev}_{c,a}$$

$$W_{c,a}^{que}=f_{c,a}^{que}\times{dev}_{c,a}$$

Using the identical deviation score to calculate the weights enables a direct comparison of the scores between the query and the reference.

The ${COUSIN}_{18}^{a}$ for each amino acid $a$ is determined by the ratio of the sum of weights of all synonymous codons associated with the amino acid in the query sequence, to the corresponding cumulative weights in the reference sequence. The ${COUSIN}_{18}$ for query sequence is obtained by summing the individual ${COUSIN}_{18}^{a}$ scores of all amino acids.

$${COUSIN}_{18}^{a}=\frac{1}{N}\times\frac{\sum_{c\in a} W_{c,a}^{que}}{\sum_{c\in a} W_{c,a}^{ref}}$$

$${COUSIN}_{18}=\sum_{a\in A} {COUSIN}_{18}^{a}$$

where $N$ represents the count of amino acids present in both the query and the reference, and $A$ denotes the set of these amino acids.

The ${COUSIN}_{59}^{a}$ for each amino acid $a$ is a weighted average of the ratio of query and reference. The ${COUSIN}_{59}$ for query sequence is obtained by summing the individual ${COUSIN}_{59}^{a}$ scores of all amino acids:

$${COUSIN}_{59}^{a}=f_{a}^{que}\times\frac{\sum_{c\in a} W_{c,a}^{que}}{\sum_{c\in a} W_{c,a}^{ref}}$$

$${COUSIN}_{59}=\sum_{a\in A} {COUSIN}_{59}^{a}$$

### Codon Bias Index (CBI)

The Codon Bias Index (CBI)^18^ provides insights into the presence of components with high CUB within a specific gene. It can be employed to characterize the expression of foreign genes in a host organism. CBI quantifies the usage of optimal codons by calculating the ratio of optimal codons to the total number of codons in a gene, with the expected usage serving as a scaling factor. A value of 1 denotes the exclusive utilization of preferred codons, whereas a value of 0 signifies random codon selection. Negative values indicate a higher frequency of nonpreferred codons being employed.

The formula for calculating CBI is as follows:

$$CBI=\frac{N_{pfr}-N_{rand}}{N_{tot}-N_{rand}}$$

where $N_{pfr}$ represents the total number of occurrences of preferred codons, $N_{rand}$ denotes the expected number of preferred codons if all synonymous codons were used equally, and $N_{tot}$ corresponds to the total number of codons in the sequence. These quantities can be calculated as follows:

$$N_{pfr}=\sum_{c\in C_{prf}} N_{c}$$

$$N_{rand}=\sum_{a\in A} N_{a}\frac{O_{a}^{prf}}{K_{a}}$$

$$N_{tot}=\sum N_{c}$$

where $C_{prf}$ represents the subset of optimal codons selected from all codons $C$ included in the analysis. $N_{c}$ refers to the number of occurrences of codon $c$ in the sequence, while $N_{a}$ represents the number of occurrences of amino acid $a$. $O_{a}^{prf}$ quantifies the number of instances where optimal codons are used for amino acid $a$, and $K_{a}$ reflects the redundancy of amino acid $a$. On our website, we defined optimal codons as the codons with the highest frequency of occurrence for each amino acid in the reference. Moreover, methionine (M) and tryptophan (W) are not involved in the calculation.

### Mean Dissimilarity-based Index (Dmean)

The Mean Dissimilarity-based Index (Dmean)^19^ is a measure that quantifies the level of diversity in synonymous codon usage across different gene sets or genomes. Dmean can also be used to analyze other compositional features, such as amino acid usage and dinucleotide relative abundance, as a genomic signature. By utilizing Dmean, we can gain a better understanding of the diversity in composition among genes. The value of Dmean ranges from 0 to 2. When all genes exhibit identical synonymous codon preferences for all amino acids, Dmean will be at its minimum value of 0. In simpler terms, lower Dmean values indicate a lower level of diversity in the usage of codons. This measure allows researchers to compare and rank different genomes based on their overall codon usage diversity, which can be calculated as follows:

$$Dmean=\frac{\sum D\left( X_{i},X_{j} \right)}{N\left( N-1 \right)/2}$$

where $N$ is the total number of genes, and $D\left( X_{i},X_{j} \right)$ is the pearson correlation distance, which can be employed to measure the dissimilarity in synonymous codon usage between two genes denoted as $X_{i}$ and $X_{j}$. This distance metric is computed as one minus the Pearson's product moment correlation coefficient $cor\left( X_{i},X_{j} \right)$:

$$D\left( X_{i},X_{j} \right)=1-cor\left( X_{i},X_{j} \right)$$

The $cor\left( X_{i},X_{j} \right)$ is a widely used measure that quantifies the linear relationship between two genes, and can be calculated as the ratio of the covariance between $X_{i}$ and $X_{j}$ to the product of their standard deviations:

$$cor\left( X_{i},X_{j} \right)=\frac{cov\left( X_{i},X_{j} \right)}{\sigma\left( X_{i} \right)\cdot\sigma\left( X_{j} \right)}=\frac{\sum_{i=1}^{n} \left( X_{i}-\bar{X_{j}} \right)\left( X_{i}-\bar{X_{j}} \right)}{\left| X_{i}-\bar{X_{j}} \right|\cdot\left| X_{i}-\bar{X_{j}} \right|}$$

This coefficient ranges from -1 to 1. A positive value indicates a direct or positive relationship, whereas a negative value indicates an inverse or negative relationship. A value close to 0 suggests a weak or negligible linear correlation. Therefore, the $D\left( X_{i},X_{j} \right)$ can range from 0 to 2. A value of 0 indicates the maximum similarity between two genes in terms of synonymous codon usage, while a value of 2 represents the maximum diversity.

Moreover, $X_{i}$ and $X_{j}$ in the $i$-th and $j$-th coding sequence is two collections of $x_{ac}$, which consists of 59 codons (methionine, tryptophan, and stop codons were excluded). The value of $x_{ac}$ can be calculated as follows:

$$x_{ac}=\frac{n_{ac}}{max\left( n_{ac} \right)}$$

where $n_{ac}$ represents the number of occurrences of $c$-th codon for the $a$-th amino acid, and $max\left( n_{ac} \right)$ represents the number of occurrences of the most frequently used synonymous codon for the same amino acid.

On our website, the Dmean is calculated as the Pearson correlation distance between the reference gene and the query gene. Lower values indicate a greater similarity in the pattern of codon usage between the reference and the query gene, as well as higher levels of gene expression.

### Relative Codon Adaptation (RCA)

For any given reference set, the Relative Codon Adaptation (RCA)^20^ first calculates the expected frequency of a codon based on its positional base frequencies and then quantifies codon adaptation by comparing the observed codon frequency to the expected codon frequency. Consequently, RCA explicitly takes into account the genomic base composition, enabling more reliable and precise estimates of gene expression, and a higher RCA score for genes that tend to include codons that are more frequent in highly expressed genes. This enhanced accuracy is particularly noticeable in scenarios with high mutational bias or reduced selection for translational efficiency, where CAI might be susceptible to misinterpretation due to mutational bias artifacts in the reference set.

Similar to CAI and RCBS, the score of RCA in the gene is calculated as the geometric mean of the ${RCA}_{xyz}$ values for each codon $xyz$:

$$RCA=\left( \prod_{k=1}^{L} {RCA}_{xyz\left( k \right)} \right)=\mathrm{ex}p \left( \frac{1}{L}\sum_{k=1}^{L} {RCA}_{xyz\left( k \right)} \right)$$

where $L$ represents the length of the query sequence measured in codons and ${RCA}_{xyz\left( k \right)}$ is the RCA value for the $k$-th codon in the gene, which can be calculated as follows:

$${RCA}_{xyz}=\frac{f\left( x,y,z \right)}{f_{1}\left( x \right)f_{2}\left( y \right)f_{3}\left( z \right)}$$

where $f\left( x,y,z \right)$ represents the observed frequency of codon $xyz$ in the reference, and x, y, and z denote the first, second, and third nucleotides of that codon, respectively. Additionally, $f_{1}\left( x \right)$, $f_{2}\left( y \right)$ and $f_{3}\left( z \right)$ represent the observed frequencies of the individual bases $x$, $y$, and $z$ at positions 1, 2, and 3 of the codons in the same reference set.

### Codon Usage Frequency Similarity (CUFS)

The Codon Usage Frequency Similarity (CUFS)^21^ is a novel measure of functional and expression similarity between genes that incorporates the frequency of codons and reflects similarities in amino acid usage. By examining the codon bias and its connection to gene expression regulation, CUFS becomes a valuable tool for understanding gene function. Despite considering post-transcriptional aspects and genomic organization, CUFS demonstrates a strong correlation with 3D genomic distance, highlighting its significance in assessing functional similarity between genes.

The CUFS offers a continuous metric based on Endres–Schindelin (ES) metric^32^ to evaluate the degree of similarity between genes and returns a distance estimation. Specifically, as genes demonstrate greater resemblance in terms of codon usage frequencies, their inferred distance is substantially reduced, resulting in lower CUFS values. In other words, a lower CUFS value indicates greater resemblance in terms of codon usage frequencies. A value of 0 indicates that the codon usage frequency between two genes is completely identical. Given the frequency vectors of a gene pair $p$ and $q$, the calculation of Codon Usage Frequency (CUF) distance(similarity) between them, utilizing the ES metric, is as follows:

$$m=\frac{1}{2}\left( p+q \right)$$

$$d_{ES}\left（ p,q \right）=\sqrt{d_{KL}\left（ p,m \right）+d_{KL}\left（ q,m \right）}$$

where $d_{KL}$ is Kullback-Leibler (KL)^33^ divergence, also known as relative entropy, which is a measure used to quantify the amount of information lost when one probability distribution is used to approximate another. It calculates the average difference in information content between corresponding elements of the two distributions. When the KL divergence is 0, it signifies that the two distributions are completely identical. Mathematically, KL divergence is defined as follows:

$$d_{KL}\left（ p,q \right）=\sum p_{i}log\frac{p_{i}}{q_{i}}$$

The frequency vectors of codon usage for the same amino acid can be computed as follows:

$$c_{i}=\frac{n_{i}}{\sum_{j\in AA} n_{j}}$$

$$\sum_{i=1}^{61} c_{i}=20$$

where the number of observed codons ($n_{i}$) is normalized by dividing it by the sum of all synonymous codons encoding the same amino acid.

### Codon Usage Bias (B)

The Codon Usage Bias (B)^22^ is a metric derived from the frequency-weighted sum of distances between the relative codon usage frequencies of two sets of genes. It is utilized to estimate the expression level by comparing the fraction of the distance of the query set concerning all genes to the distance from a reference set or a linear combination of reference sets.

When considering $F$ as a particular gene and $R$ as the set of all genes (reference), the codon bias of $F$ with respect to $R$ can be calculated using the following formula:

$$B\left( F\left| R \right. \right)=\sum_{a} P_{a}\left( F \right)\sum_{xyz\in a} \left| f_{xyz}-r_{xyz} \right|$$

where $f_{xyz}$ and $r_{xyz}$ are the observed frequency of codon $xyz$, normalized by dividing each by the sum of all synonymous codons encoding the same amino acid. For each amino acid $a$, $\sum_{xyz\in a} f_{xyz}=1$ and $\sum_{xyz\in a} r_{xyz}=1$. $P_{a}\left( F \right)$ represents the set of normalized amino acid frequencies of the gene $F$, and $\sum_{a} P_{a}\left( F \right)=1$.

Similarly, the codon bias of $R$ with respect to $F$ is calculated as follows:

$$B\left( R\left| F \right. \right)=\sum_{a} P_{a}\left( R \right)\sum_{xyz\in a} \left| r_{xyz}-f_{xyz} \right|$$

where $P_{a}\left( R \right)$ represents the set of normalized amino acid frequencies of the gene $R$, and $\sum_{a} P_{a}\left( R \right)=1$.

For $B\left( F\left| R \right. \right)$ and $B\left( R\left| F \right. \right)$, the maximum possible value is 2.00 and uncommon to exceed 0.5. Codon usage differences between two gene families generally range from 0.05 to 0.300. A higher value indicates significant codon usage differences between the two sets of genes. A symmetric version of $B$ is obtained by averaging $B\left( F\left| R \right. \right)$ and $B\left( R\left| F \right. \right)$:

$$B=\left( B\left( F\left| R \right. \right)+B\left( R\left| F \right. \right) \right)/2$$

In general, the value of $B\left( F\left| R \right. \right)$ and $B\left( R\left| F \right. \right)$ differ little, indicating that differences in amino acid usages between $F$ and $R$ have little influence on the calculated relative codon biases. Consequently, we have opted to adopt the $B\left( F\left| R \right. \right)$ version as the ultimate outcome.

## Indices based on adaptation to the tRNA levels and their supply

Indices in this section are based on the adaptation of codons to the levels of tRNA in the cell. tRNA molecules are considered as major factors that influence translation elongation at the genomic level. Previous research has indicated that intracellular tRNA levels are correlated with codon usage and amino acid composition in various prokaryotic and eukaryotic species^34,35^. It is widely accepted that the preference for certain codons is due to differences in the abundance of corresponding tRNAs within the cell. Generally, codons with higher tRNA abundance are utilized more frequently. Genes that employ these preferred codons tend to exhibit enhanced translation efficiency and accuracy.

### tRNA Adaptation Index (tAI)

The tRNA Adaptation Index (tAI)^23^ is a quantifiable metric used to assess translational efficiency by considering the intracellular abundance of tRNA molecules and the efficacy of each codon-anticodon interaction. The tAI value ranges from 0 to 1, with higher values indicating that genes tend to incorporate codons that are more adapted to the tRNA pool. Specifically, the index incorporates weights that have been initially derived from gene expression data in Saccharomyces cerevisiae. These weights assign values to each wobble interaction, reflecting the efficiency of the codon-anticodon pairing during translation, which can be calculated as follows:

$$W_{i}=\sum_{j=1}^{n_{i}} \left( 1-s_{ij} \right){tGCN}_{ij}$$

where $n_{i}$ is the number of tRNA types/anticodons that pair with the ith codon, ${tGCN}_{ij}$ is the gene copy number of the $j$-th tRNA molecule that recognizes the $i$-th codon. The copy numbers of tRNA can be retrieved from Genomic tRNA Database (http://gtrnadb.ucsc.edu/). The $s_{ij}$ (**Supplementary Table 4**) represents the selective constraint governing the efficiency of the codon-anticodon coupling. This value falls within the range of 0 to 1, with values closer to 0 indicating a more efficient wobble interaction between the codon and anticodon.

**Supplementary Table 4** The optimized s-values values of the codon-anticodon coupling:

| S | dosReis^23^ | Tuller^36^ |
| --- | --- | --- |
| S_G:U_ | 0.41 | 0.561 |
| S_I:C_ | 0.28 | 0.28 |
| S_I:A_ | 0.9999 | 0.9999 |
| S_U:G_ | 0.68 | 0.68 |
| S_L:A_ | 0.89 | 0.89 |

L, lysidine; I, inosine. The values of dosReis and Tuller can refer to literature^23^, ^36^. In this work, we use the s-values measured by dosReis et al.

To determine the classical translational effiency ${cTE}_{i}$ of a codon *i*, normalized weights can be utilized. These normalized weights can be obtained by the following formula to have a maximum value of 1:

$${cTE}_{i}=\left\{ \begin{aligned} W_{i}/W_{max} if W_{i} \neq0 \\ W_{geomean} if W_{i} =0 \end{aligned} \right.$$

where $W_{max}$ is the maximum $W_{i}$ value and $W_{geomean}$ is the geometric mean of all ${cTE}_{i}$ with $W_{i} \neq0$.

The tAI of a gene (${tAI}_{g}$) is the geometric mean of these relative adaptiveness values, and ranges from 0 (low efficiency) to 1 (high efficiency):

$${tAI}_{g}=\left( \prod_{k=1}^{L} {cTE}_{k} \right)^{1/L}=\mathrm{ex}p \left( \frac{1}{L}\sum_{k=1}^{L} ln{cTE}_{k} \right)$$

where $L$ is the length of a gene measured in codons and ${cTE}_{k}$ is the translational effiency for the $k$-th codon in the gene.

### Genetic tRNA Adaptation Index (gtAI)

The initial implementation of the tRNA adaptation index (tAI)^23^ exhibited notable shortcomings. The $s_{ij}$ weights generated were optimized based on gene expression patterns in Saccharomyces cerevisiae, potentially leading to variations across different species. The subsequent stAI^37,38^ method utilized a hill climbing algorithm for $s_{ij}$ weight optimization, but this approach wasn't optimal for complex search spaces, potentially struggling to locate the global maximum even with varied starting points. To overcome these limitations, a species-specific approach called the Genetic tRNA Adaptation Index (gtAI)^24^ was developed.

The gtAI employs a genetic algorithm to obtain the best set of $s_{ij}$ weights, addressing the issue of obtaining meaningful weights for each organism. It also uses robust CUB indices, namely ENC^2,3^ and RSCU^1^, instead of the directional codon bias score (DCBS) ^5^ used in stAI. Highly expressed genes are influenced by translational selection, resulting in the incorporation of codons that better adapt to the intracellular tRNA pool. Therefore, a correlation is expected between RSCU and absolute adaptiveness ($W_{i}$) values. In the stAI, unique $s_{ij}$ weights are optimized for each organism by maximizing the nonparametric correlation between RSCU and $W_{i}$ using a genetic algorithm. This algorithm is a metaheuristic search approach inspired by the concept of survival of the fittest, which algorithm is as follows:

**Input:** Genome coding sequences

Initialize S, vector of the initial population as chromosomes (Sij sets) with random Sij values (genes) Generation time = n;

**For** s in S **do**

Evaluate fitness function(s);

n += 1

IntialLabel;

**Test:**

Selection(s) where Sij sets that exhibit higher correlation between RSCU and Wi;

**Do:**

Crossover(s);

Mutation(s);

Evaluate fitness function(s);

**If** n = Generation time, **then**

Output = Best fitness(s);

**Else**

Go to IntialLabel

**Output:** the best set of Sij weights + tAI values

In this work, the genetic algorithm operates with a population size of 60 and runs for 1000 iterations to search for the best $s_{ij}$ weights that maximize the correlation between RSCU and $W_{i}$. Then, the best set of $s_{ij}$ weights will be used to calculate the genetic tRNA adaptation index (gtAI) using the following equations:

$$W_{i}=\sum_{j=1}^{n_{i}} \left( 1-s_{ij} \right){tGCN}_{ij}$$

$$gtAI=\left( \prod_{k=1}^{L} W_{k} \right)^{1/L}=\mathrm{ex}p \left( \frac{1}{L}\sum_{k=1}^{L} lnW_{k} \right)$$

where $L$ is the length of a gene measured in codons and $W_{k}$ is the weight for the $k$-th codon in the gene. Same as tAI, the value of gtAI ranges from 0 to 1, with higher values indicating that genes tend to incorporate codons that are more adapted to the tRNA pool.

### P2 Index

The P2 Index^25^ quantifies the efficiency of interactions between codons and their corresponding anticodons, offering insights into translation efficiency in cases where information regarding preferred codon sets is unavailable. A higher P2 value is typically observed in highly expressed genes, whereas genes with lower expression levels tend to exhibit lower P2 values. Furthermore, a P2 value exceeding 0.5 indicates the presence of translational selection influencing the coding sequence. P2 Index is derived from the proportion of pyrimidine-ending codons that exhibit intermediate strength, which can be calculated according to the following equation:

$$P2=\frac{WWC+SSU}{WWY+SSY}$$

where W = A or U, S = C or G, and Y = C or U. WWC, SSU, WWY and SSY represent the frequency of their corresponding codons, respectively.

## Indices based on complex statistical patterns of codon usage

Indices in this section are based on measures of complex patterns of codon usage. In the coding region, sequences longer than a single codon may contain regulatory codes that pertain to various aspects of gene expression and intracellular processes. Therefore, it becomes necessary to develop indices that can capture these aspects, which cannot be adequately computed based solely on single codon distributions. Changes in the composition of codons can have an impact on these longer patterns, inducing a selection pressure on codon composition. Consequently, the codon-based indices should encompass measures that go beyond analyzing single codons and instead employ more advanced statistical methods. Indices in this group capture complex signals that are influenced by and, in turn, influence codons.

### GC Content at the First Position of Synonymous Codons (GC1)

The GC Content at the First Position of Synonymous Codons (GC1) represents the frequency of G+C usage at the first position of synonymous codons, which can be calculated as follows:

$$GC1=\frac{GNN+CNN}{ANN+TNN+GNN+CNN}$$

where $N$ represent an arbitrary base, and $ANN$, $TNN$, $GNN$ and $CNN$ represent the frequency of their corresponding codons, respectively.

### GC Content at the Second Position of Synonymous Codons (GC2)

The GC Content at the Second Position of Synonymous Codons (GC2) represents the frequency of G+C usage at the second position of synonymous codons, which can be calculated as follows:

$$GC2=\frac{NGN+NCN}{NAN+NTN+NGN+NCN}$$

where $N$ represent an arbitrary base, and $NAN$, $NTN$, $NGN$ and $NCN$ represent the frequency of their corresponding codons, respectively.

### GC Content at the Third Position of Synonymous Codons (GC3)

The GC Content at the Third Position of Synonymous Codons (GC3)^26^ represents the frequency of G+C usage at the third position of synonymous codons, which exhibits variability in nucleotide composition. Changes in the third position of codons typically do not result in alterations to the encoded amino acids. Furthermore, base mutations occurring at this position are often subjected to less selection pressure. Consequently, the investigation of the base composition at position 3 holds significance in the study of codon preference. GC3 ranges from 0 to 1, and a higher value is correlated with highly expressed genes.

The formula for GC3 is as follows:

$$GC3=\frac{NNG+NNC}{NNA+NNT+NNG+NNC}$$

where $N$ represent an arbitrary base, and $NNA$, $NNT$, $NNG$ and $NNC$ represent the frequency of their corresponding codons, respectively.

### GC Content (GC)

The GC content, representing the percentage of guanine (G) and cytosine (C) nitrogenous bases in a gene, is a valuable measure for assessing the base composition. This measure provides insights into the relative proportion of G and C bases compared to the other two bases, adenine (A) and thymine (T) in DNA, or adenine (A) and uracil (U) in RNA. The formula for GC3 is as follows:

$$GC=\frac{G+C}{A+T+G+C}$$

where $A$, $T$, $G$, $C$ represent the number of corresponding bases in the gene, respectively.

Although AT-rich intergenic regions can reduce the overall GC content of a gene or genome, there exists a strong correlation between the local GC composition (GC1, GC2, and GC3) and the overall GC composition of the sequence. In fact, the higher the overall GC content bias, the higher the local GC composition.

### Effective Number of Codon Pairs (ENcp)

The Similar to ENC, Effective Number of Codon Pairs (ENcp)^27^ is another metric used to quantify codon usage bias in a genome. By comparing the observed frequencies of codon pairs to the expected frequencies under the assumption of equal codon pair usage, ENcp provides a numerical value that reflects the level of bias or preference in codon pair usage. ENcp can range from 20 (when no bias or all the synonymous codon pairs are used uniformly) to 61 (when the maximal bias or preference observed in synonymous codon pair usage). The calculation of ENcp is analogous to that of ENC, with the additional incorporation of a square root:

$$\hat{N}_{cp}=\sqrt{\sum_{m} \frac{k_{m}}{\bar{F}_{m}}}$$

where $\bar{F}_{m}$ represents the average value of $F_{AA}$ for all amino acid pairs with degeneracy $m$, and $k_{m}$ denotes the count of amino acid pairs that share $m$ synonymous representations.

For standard genetic code, ENcp can be calculated as follows:

$$\hat{N}_{cp}=\sqrt{4+\frac{36}{\bar{F}_{2}}+\frac{4}{\bar{F}_{3}}+\frac{101}{\bar{F}_{4}}+\frac{30}{\bar{F}_{6}}+\frac{90}{\bar{F}_{8}}+\frac{1}{F_{9}}+\frac{64}{\bar{F}_{12}}+\frac{25}{\bar{F}_{16}}+\frac{6}{\bar{F}_{18}}+\frac{30}{\bar{F}_{24}}+\frac{9}{\bar{F}_{36}}}$$

### Codon Pair Score (CPS)

The Codon Pair Score (CPS)^28,29^ is a metric used to assess the similarity in codon pair preferences between viruses and their host species. It is calculated by taking the natural logarithm of the observed ratio of a specific codon pair's occurrence to the expected number of occurrences of that codon pair in all protein-coding sequences of a species:

$${CPS}_{i}=ln\left( \frac{F\left( AB \right)}{\frac{F\left( A \right)\cdot F\left( B \right)}{F\left( X \right)\cdot F\left( Y \right)}\cdot F\left( XY \right)} \right)$$

where $F\left( AB \right)$ is the frequency of a specific codon pair $AB$, $F\left( A \right)$ and $F\left( B \right)$ represent the frequencies of individual codons $A$ and codon $B$, respectively. Similarly, $F\left( XY \right)$ is the frequency of an amino acid pair $XY$, $F\left( X \right)$ and $F\left( Y \right)$ represent the frequencies of individual amino acids $X$ and amino acid $Y$, respectively. A positive CPS value signifies that the given codon pair is statistically over-represented, while a negative CPS indicates that the pair is statistically under-represented. In other words, a higher positive CPS score signifies a stronger preference for a specific codon pair. In addition, Codon pairs that are equally under or overrepresented have a CPS value equidistant from 0.

The average CPS values of all codon pairs present in a gene are collectively referred to as Codon Pair Bias (CPB), which can be calculated as follows:

$$CPB=\sum_{k=1}^{L} \frac{{CPS}_{k}}{L-1}$$

where $L$ is the length of a gene and ${CPS}_{k}$ is the CPS value for the $k$-th codon in the gene.

### Codon Volatility

The Codon Volatility^30^ refers to the probability of a nonsynonymous change occurring through a random point mutation in the codon. The volatility of a codon is influenced by mutational patterns, including the ratio of transitional to transversional changes. In the simplest mutation model (SMM), where all nucleotides have equal mutation rates and are equally exchangeable, codon volatility is determined by the proportion of neighboring codons that encode different amino acids due to point mutations. For example, a codon such as TTG (encoding Leucine) has a volatility of 6/8, as 6 out of its 8 neighboring codons (excluding stop codons) lead to nonsynonymous changes. The range of codon volatility spans from 0.5 (e.g., CGA for Arginine) to 1 (e.g., TGG for Tryptophan or ATG for Methionine). The volatility of a codon c is employed as a measure to quantify the likelihood that the most recent nucleotide mutation in that codon resulted in an amino acid substitution. If a gene contains numerous residues that are under selective pressure for amino acid replacements, the resulting codons within that gene will, on average, display increased volatility. Conversely, if a gene is subjected to strong purifying selection to maintain its amino acids, the resulting sequence will, on average, exhibit lower volatility.

The codon volatility is calculated as follows:

$$v\left( c \right)=\frac{1}{no. of neighbours}\sum_{neighbours c_{i}} D\left( acid\left( c \right),acid\left( c_{i} \right) \right)$$

where $no. of neighbours$ is for the sum of sense codon $c_{i}$ that can mutate into $c$ by a single point mutation. $D$, representing the Hamming distance, is defined as zero when two amino acids are identical and one otherwise.

The volatility of a gene can be computed as the arithmetic of codon volatility, which can be calculated as follows:

$$v\left( g \right)=\frac{\sum_{k=1}^{L} v_{k}}{L}$$

where $L$ is the length of a gene measured in codons and $v_{k}$ is the volatility for the $k$-th codon in the gene.

# Analysis

By comparing the Spearman correlations of these indices for protein expression across different species (**Supplementary Table 5**), this work highlights the significance of species-specific differences when selecting appropriate indices for research studies.

| **Supplementary Table 5 Absolute Spearman correlation coefficients between protein expression and indices** | | | | |
| --- | --- | --- | --- | --- |
|  | **Saccharomyces cerevisiae** | **Cricetulus griseus** | **Escherichia coli** | **Mus musculus** |
| **RSCU** | 0.405 | 0.073 | 0.389 | 0.127 |
| **ENC** | 0.390 | 0.028 | 0.395 | 0.091 |
| **RCBS** | 0.360 | 0.122 | 0.399 | 0.140 |
| **DCBS** | 0.390 | 0.049 | 0.418 | 0.137 |
| **CDC** | 0.386 | 0.026 | 0.398 | 0.128 |
| **MILC** | 0.288 | 0.220 | 0.300 | 0.028 |
| **ICDI** | 0.315 | 0.159 | 0.378 | 0.131 |
| **SCUO** | 0.360 | 0.102 | 0.382 | 0.135 |
| **Ew** | 0.360 | 0.102 | 0.382 | 0.135 |
| **P** | 0.410 | 0.111 | 0.381 | 0.146 |
| **MCB** | 0.380 | 0.087 | 0.320 | 0.084 |
| **CAI** | 0.362 | 0.234 | 0.187 | 0.096 |
| **FOP** | 0.307 | 0.202 | 0.118 | 0.085 |
| **COUSIN59** | 0.021 | 0.272 | 0.083 | 0.074 |
| **COUSIN18** | 0.011 | 0.248 | 0.026 | 0.067 |
| **CBI** | 0.307 | 0.232 | 0.154 | 0.080 |
| **Dmean** | 0.061 | 0.323 | 0.309 | 0.037 |
| **RCA** | 0.448 | 0.187 | 0.173 | 0.028 |
| **CUFS** | 0.244 | 0.361 | 0.371 | 0.133 |
| **B** | 0.236 | 0.328 | 0.391 | 0.115 |
| **tAI** | 0.547 | 0.033 | 0.375 | 0.137 |
| **gtAI** | 0.507 | 0.077 | 0.407 | 0.141 |
| **p2 index** | 0.383 | 0.099 | 0.400 | 0.034 |
| **GC** | 0.194 | 0.300 | 0.161 | 0.037 |
| **GC1** | 0.273 | 0.150 | 0.055 | 0.007 |
| **GC2** | 0.033 | 0.320 | 0.120 | 0.007 |
| **GC3** | 0.080 | 0.222 | 0.160 | 0.070 |
| **ENCP** | 0.196 | 0.112 | 0.077 | 0.100 |
| **CPS** | 0.061 | 0.150 | 0.315 | 0.025 |
| **Codon volatility** | 0.087 | 0.210 | 0.037 | 0.021 |

# Summary

In summary, we have updated the rare codon analysis tool to include a wider range of indices and expression host species, enabling a more comprehensive evaluation of codon usage preferences. We have conducted thorough checks to ensure the accuracy of the calculations, providing reliable results for researchers. To the best of our knowledge, there is currently a lack of comprehensive toolkits or websites that offer species-specific CUB calculations. As such, our website is positioned to become a highly valuable and influential tool in the fields of bioinformatics, computational biology, and proteome research. Its unique capabilities make it an indispensable resource for researchers in these domains.

We are pleased to offer free access to our website (https://www.genscript.com/tools/rare-codon-analysis), where users can obtain detailed reports on the analysis of rare codons for their sequences.

# References

1. Sharp, P. M. & Li, W.-H. An evolutionary perspective on synonymous codon usage in unicellular organisms. *J. Mol. Evol.* **24**, 28–38 (1986).

2. Wright, F. The ‘effective number of codons’ used in a gene. *Gene* **87**, 23–29 (1990).

3. Satapathy, S. S., Sahoo, A. K., Ray, S. K. & Ghosh, T. C. Codon degeneracy and amino acid abundance influence the measures of codon usage bias: improved Nc (  *N̂ _c_*  ) and ENCprime (  *N̂ ^′^ _c_*  ) measures. *Genes Cells* **22**, 277–283 (2017).

4. Roymondal, U., Das, S. & Sahoo, S. Predicting Gene Expression Level from Relative Codon Usage Bias: An Application to Escherichia coli Genome. *DNA Res.* **16**, 13–30 (2009).

5. Sabi, R. & Tuller, T. Modelling the Efficiency of Codon–tRNA Interactions Based on Codon Usage Bias. *DNA Res.* **21**, 511–526 (2014).

6. Zhang, Z. *et al.* Codon Deviation Coefficient: a novel measure for estimating codon usage bias and its statistical significance. *BMC Bioinformatics* **13**, 43 (2012).

7. Supek, F. & Vlahoviček, K. Comparison of codon usage measures and their applicability in prediction of microbial gene expressivity. *BMC Bioinformatics* **6**, 182 (2005).

8. Freire-Picos, M. A. *et al.* Codon usage in Kluyveromyces lactis and in yeast cytochrome c-encoding genes. *Gene* **139**, 43–49 (1994).

9. Wan, X.-F., Xu, D., Kleinhofs, A. & Zhou, J. Quantitative relationship between synonymous codon usage bias and GC composition across unicellular genomes. *BMC Evol. Biol.* **4**, 19 (2004).

10. Wan, X.-F., Zhou, J. & Xu, D. CodonO: a new informatics method for measuring synonymous codon usage bias within and across genomes. *Int. J. Gen. Syst.* **35**, 109–125 (2006).

11. Suzuki, H., Saito, R. & Tomita, M. The ‘weighted sum of relative entropy’: a new index for synonymous codon usage bias. *Gene* **335**, 19–23 (2004).

12. Gribskov, M., Devereux, J. & Burgess, R. R. The codon preference plot: graphic analysis of protein coding sequences and prediction of gene expression. *Nucleic Acids Res.* **12**, 539–549 (1984).

13. Urrutia, A. O. & Hurst, L. D. Codon Usage Bias Covaries With Expression Breadth and the Rate of Synonymous Evolution in Humans, but This Is Not Evidence for Selection. *Genetics* **159**, 1191–1199 (2001).

14. Sharp, P. M. & Li, W.-H. The codon adaptation index-a measure of directional synonymous codon usage bias, and its potential applications. *Nucleic Acids Res.* **15**, 1281–1295 (1987).

15. Ikemura, T. Correlation between the abundance of Escherichia coli transfer RNAs and the occurrence of the respective codons in its protein genes: A proposal for a synonymous codon choice that is optimal for the E. coli translational system. *J. Mol. Biol.* **151**, 389–409 (1981).

16. Ikemura, T. Correlation between the abundance of yeast transfer RNAs and the occurrence of the respective codons in protein genes. *J. Mol. Biol.* **158**, 573–597 (1982).

17. Bourret, J., Alizon, S. & Bravo, I. G. COUSIN (COdon Usage Similarity INdex): A Normalized Measure of Codon Usage Preferences. *Genome Biol. Evol.* **11**, 3523–3528 (2019).

18. Bennetzen, J. L. & Hall, B. D. Codon selection in yeast. *J. Biol. Chem.* **257**, 3026–3031 (1982).

19. Suzuki, H., Saito, R. & Tomita, M. Measure of synonymous codon usage diversity among genes in bacteria. *BMC Bioinformatics* **10**, 167 (2009).

20. Fox, J. M. & Erill, I. Relative Codon Adaptation: A Generic Codon Bias Index for Prediction of Gene Expression. *DNA Res.* **17**, 185–196 (2010).

21. Diament, A., Pinter, R. Y. & Tuller, T. Three-dimensional eukaryotic genomic organization is strongly correlated with codon usage expression and function. *Nat. Commun.* **5**, 5876 (2014).

22. Karlin, S., Mrázek, J. & Campbell, A. M. Codon usages in different gene classes of the *Escherichia coli* genome. *Mol. Microbiol.* **29**, 1341–1355 (1998).

23. Reis, M. d. Solving the riddle of codon usage preferences: a test for translational selection. *Nucleic Acids Res.* **32**, 5036–5044 (2004).

24. Anwar, A. M. *et al.* gtAI: an improved species-specific tRNA adaptation index using the genetic algorithm. *Front. Mol. Biosci.* **10**, 1218518 (2023).

25. Gouy, M. & Gautier, C. Codon usage in bacteria: correlation with gene expressivity. *Nucleic Acids Res.* **10**, 7055–7074 (1982).

26. Stenico, M., Lloyd, A. T. & Sharp, P. M. Codon usage in *Caenorhabditis elegans* : delineation of translational selection and mutational biases. *Nucleic Acids Res.* **22**, 2437–2446 (1994).

27. Alexaki, A. *et al.* Codon and Codon-Pair Usage Tables (CoCoPUTs): Facilitating Genetic Variation Analyses and Recombinant Gene Design. *J. Mol. Biol.* **431**, 2434–2441 (2019).

28. Kunec, D. & Osterrieder, N. Codon Pair Bias Is a Direct Consequence of Dinucleotide Bias. *Cell Rep.* **14**, 55–67 (2016).

29. Coleman, J. R. *et al.* Virus Attenuation by Genome-Scale Changes in Codon Pair Bias. *Science* **320**, 1784–1787 (2008).

30. Plotkin, J. B., Dushoff, J. & Fraser, H. B. Detecting selection using a single genome sequence of M. tuberculosis and P. falciparum. *Nature* **428**, 942–945 (2004).
